# Nutritional interventions to support acute mTBI recovery

**DOI:** 10.3389/fnut.2022.977728

**Published:** 2022-10-14

**Authors:** Emma Finnegan, Ed Daly, Alan J. Pearce, Lisa Ryan

**Affiliations:** ^1^Department of Sport, Exercise and Nutrition, Atlantic Technological University (ATU), Galway, Ireland; ^2^College of Science, Health and Engineering, La Trobe University, Melbourne, VIC, Australia

**Keywords:** brain injury - traumatic, mild traumatic brain injury (mTBI), concussion, omega 3 (n-3) polyunsaturated fatty acids, vitamin, supplementation, nutrition

## Abstract

When mild traumatic brain injury (mTBI) occurs following an impact on the head or body, the brain is disrupted leading to a series of metabolic events that may alter the brain's ability to function and repair itself. These changes may place increased nutritional demands on the body. Little is known on whether nutritional interventions are safe for patients to implement post mTBI and whether they may improve recovery outcomes. To address this knowledge gap, we conducted a systematic review to determine what nutritional interventions have been prescribed to humans diagnosed with mTBI during its acute period (<14 days) to support, facilitate, and result in measured recovery outcomes.

**Methods:**

Databases CINAHL, PubMed, SPORTDiscus, Web of Science, and the Cochrane Library were searched from inception until January 6, 2021; 4,848 studies were identified. After removing duplicates and applying the inclusion and exclusion criteria, this systematic review included 11 full papers.

**Results:**

Patients that consumed enough food to meet calorie and macronutrient (protein) needs specific to their injury severity and sex within 96 h post mTBI had a reduced length of stay in hospital. In addition, patients receiving nutrients and non-nutrient support within 24–96 h post mTBI had positive recovery outcomes. These interventions included omega-3 fatty acids (DHA and EPA), vitamin D, mineral magnesium oxide, amino acid derivative *N*-acetyl cysteine, hyperosmolar sodium lactate, and nootropic cerebrolysin demonstrated positive recovery outcomes, such as symptom resolution, improved cognitive function, and replenished nutrient deficiencies (vitamin D) for patients post mTBI.

**Conclusion:**

Our findings suggest that nutrition plays a positive role during acute mTBI recovery. Following mTBI, patient needs are unique, and this review presents the potential for certain nutritional therapies to support the brain in recovery, specifically omega-3 fatty acids. However, due to the heterogenicity nature of the studies available at present, it is not possible to make definitive recommendations.

**Systematic review registration:**

The systematic review conducted following the PRISMA guidelines protocol was registered (CRD42021226819), on Prospero.

## Introduction

Mild traumatic brain injury (mTBI) is responsible for up to 90% of all traumatic brain injuries (TBI) globally (1–3). However, this figure is likely to be underestimated because many mTBIs go undiagnosed or unreported (1–4). Mild TBI is identified by an impact, directly or indirectly to the head from an external physical force (3–5), with a Glasgow Coma Scale (GCS) score between 13 and 15. In addition, various physical (e.g., a loss of coordination and/or vomiting) and cognitive (e.g., confusion and/or memory loss) impairments may occur, along with temporary amnesia (6–8).

These cognitive, sensory, behavioral, emotional, and physical disturbances following mTBI affect functioning of the brain and may lead to long-term impairments if not treated and managed correctly (3, 6, 9–13). It may take time for injury symptoms to manifest, and severity will vary. Nevertheless, most individuals should recover within 1–3 months (14, 15). Incidence of mTBI can occur following a wide range of events, from a motor vehicle accident, a fall, assault, a military blast, as well as collisions in sport, or children playing. As a result, mTBI affects people of all ages and sex (4, 6, 16, 17).

In response to mTBI, a cascade of secondary neurometabolic events occur which can create functional disturbances, imbalances between cellular ions (Na^+^, K^+^ and Ca^2+^), an overproduction of free radicals, and result in an “energy crisis” (18). During the “energy crisis,” metabolic homeostasis, energy metabolism, and blood flow processes are disrupted. This energy supply and demand mismatch results in hypermetabolic events such as hyperglycemia, protein catabolism, and as a consequence, result in a hypometabolic cellular states (18, 19). The intracellular flux of ions (Ca^2+^) contributes to mitochondrial dysfunction, oxidative stress, neuroinflammation, cellular damage and in some cases death (7, 12, 19, 20). Romeu-Mejia et al. (12) provide a detailed account of TBI pathophysiology; consequent neuroinflammation, blood-brain barrier disruption, cell membrane damage and death (12).

During the secondary phase of injury, free radical production, oxidation, and inflammation accelerate aiming to protect the brain, restore functioning and undo intracellular damage (20, 21). As a result, these changes increase the brain's need for anti-inflammatory and antioxidant nutrients. However, oxidation can become damaging and deplete cellular antioxidant levels if prolonged; promoting further metabolic disruption and neuroinflammation, which is associated with the worsening of symptoms, their duration, and the risk of developing persistent post-concussion syndrome (PCS) (20, 22). Inflammatory responses will be initiated to provide neuroprotection but, if prolonged, will hinder brain recovery and result in PCS (18, 23, 24). Therefore, if not correctly managed these originally positive mechanisms (free radical production and inflammation) could create an environment for a secondary impact to occur, poor recovery, tissue damage and subsequent cell death - a concern for cases that go undiagnosed (19, 25). These metabolic changes, as a result, will significantly increase patients' energy and nutritional demands following an impact resulting in mTBI.

Following mTBI the majority of patients will recover. Recovery is defined clinically through the resolution of symptoms (*via* SCAT5 and symptom severity scores), illustrating cognitive and physical functioning, which will permit them to begin returning to normal daily activities, such as school, work, and sport (8, 14, 26–28). However, this does not account for neurobiological recovery. Individual symptoms are thought to resolve within 10–14 days (8, 29), however research has found that less than half of patients do so with a majority recovering from day 28 (27, 30) to 33 (31, 32) post mTBI. Present mTBI recovery protocol advice for patients is to follow a period of cognitive and physical rest for 24–48 hours (h), to improve symptoms and reduce metabolic brain demands. Following this initial rest, patients are encouraged to gradually resume normal daily cognitive and physical activities (including screen time) at a pace that does not worsen or generate new symptoms (8, 26, 31, 32). At this moment, mTBI treatments and therapy recommendations are limited. In addition, the present 2016 consensus sports statement (8) provides little evidence on using pharmacological agents or medications and at present does not advise implementing nutrition as support or strategy for acute mTBI recovery (7, 8, 25, 27, 31, 32).

A significant body of scientific knowledge is published on the role of nutritional strategies in optimizing brain development and supporting repair and function throughout life (33–37). In addition, a patient's initial nutritional intake and diet quality will influence the availability of energy and nutrients to mediate metabolic alterations and provide support to the brain post-injury (21); highlighting the role nutritional protocols play in supporting acute repair and recovery (<14 days approx.), and potentially prevent the prolonging and worsening of outcomes post mTBI (11, 38–40). However, research is limited on what nutritional support(s) is safe and effective for humans (19, 41–43) to implement post mTBI. Instead, research using nutrient and non-nutrient therapies for acute mTBI recovery has been carried out in preclinical studies and animal-based (7, 40) trials using enhanced feeding or supplement protocols. Studies have found water-soluble vitamins B and C and fat-soluble vitamins E and D, omega-3 fatty acids, minerals zinc and magnesium, curcumin, melatonin, and enzogenol effective (19, 20, 25, 43–47) in improving neurological, cognitive, and molecular recovery outcomes.

Therefore, this systematic review aimed to address this gap, to examine the current evidence on nutritional interventions prescribed to humans who have been clinically diagnosed with mTBI during its acute period (<14 days) to support, facilitate, and result in a measured outcome(s) of recovery.

## Methods

This review was conducted following the Preferred Reporting Items for Systematic Reviews and Meta-Analyses (PRISMA) guidelines (48) and registered on the International Prospective Register of Systematic Reviews (PROSPERO CRD 42021226819). The review question, Population, Intervention, Comparison, Outcomes and Study (PICOS), inclusion and exclusion criteria, and search terms are presented in Table 1 and were used to support the review, provide transparency, and minimize bias.

**Table 1:** PICOS-model and Medline search strategy in accordance with PRISMA statement.

**Primary review question/aim**	
In all humans diagnosed with concussion/mTBI, what nutrition or nutritional interventions have been prescribed during the acute phase (<14 days) following injury and resulting in recovery outcomes?	
**Inclusion criteria**	
Population	Humans all ages (children, adolescents <18 years and adult populations >18 years) clinically diagnosed with a concussion/mTBI (GCS of 13 to 15), due to any known/reported mechanism
Intervention	Following diagnosis of concussion/mTBI during the acute phase (<14 day window), either nutrition/nutritional intervention are prescribed. For this review the acute phase of injury will be defined as minutes after the event up to and including 7 days post event. All reported concussion/mTBI mechanisms
Outcomes	Measured concussion/mTBI recovery <14 days for adults and <28 days for children post injury onset. Return to play, return to activity or a clinical diagnosis of recovery
Study design	Published original research, randomized control trial (RTC), systematic reviews. Retrospective data analysis, cross sectional study design, parallel studies, where data meeting the PICO can be extracted. Abstracts (with data) will be included initially. Publications in the English language only
**Exclusion criteria**	
Population	Non-human, animals, cells and models
Intervention	Non nutritional interventions. Preclinical/animal/cell interventions. No measured or hazardous protocols in place.
Outcomes	No measure of recovery post TBI
Study design	All other study designs. Case reports, editorials, commentary's, review articles (in the case of systematic reviews if relevant data cannot be extracted or does not meet PICOS), consensus statements, positional statements, and opinion pieces, and non-English publications
**Search terms^†^**	
Preformed in Cochrane, CINAHL, PubMed, Sports discus and Web of Science databases	

† [(concussion OR mild traumatic brain injury OR mild tbi OR mtbi OR mild brain injury)* AND (diet OR food OR beverage OR calorie OR macronutrient OR micronutrient OR protein OR carbohydrate OR fat OR supplement OR antioxidant OR vitamin OR mineral OR amino acid OR fatty acid OR glucose OR creatine OR nutri OR nutraceutical OR keto OR omega 3 OR Docosahexaenoic acid OR DHA OR herb)* AND (recovery OR return to play OR rehabilitation)*].

^*^Keywords were the same for each database searched.

### Search strategy

**Figure 1:**
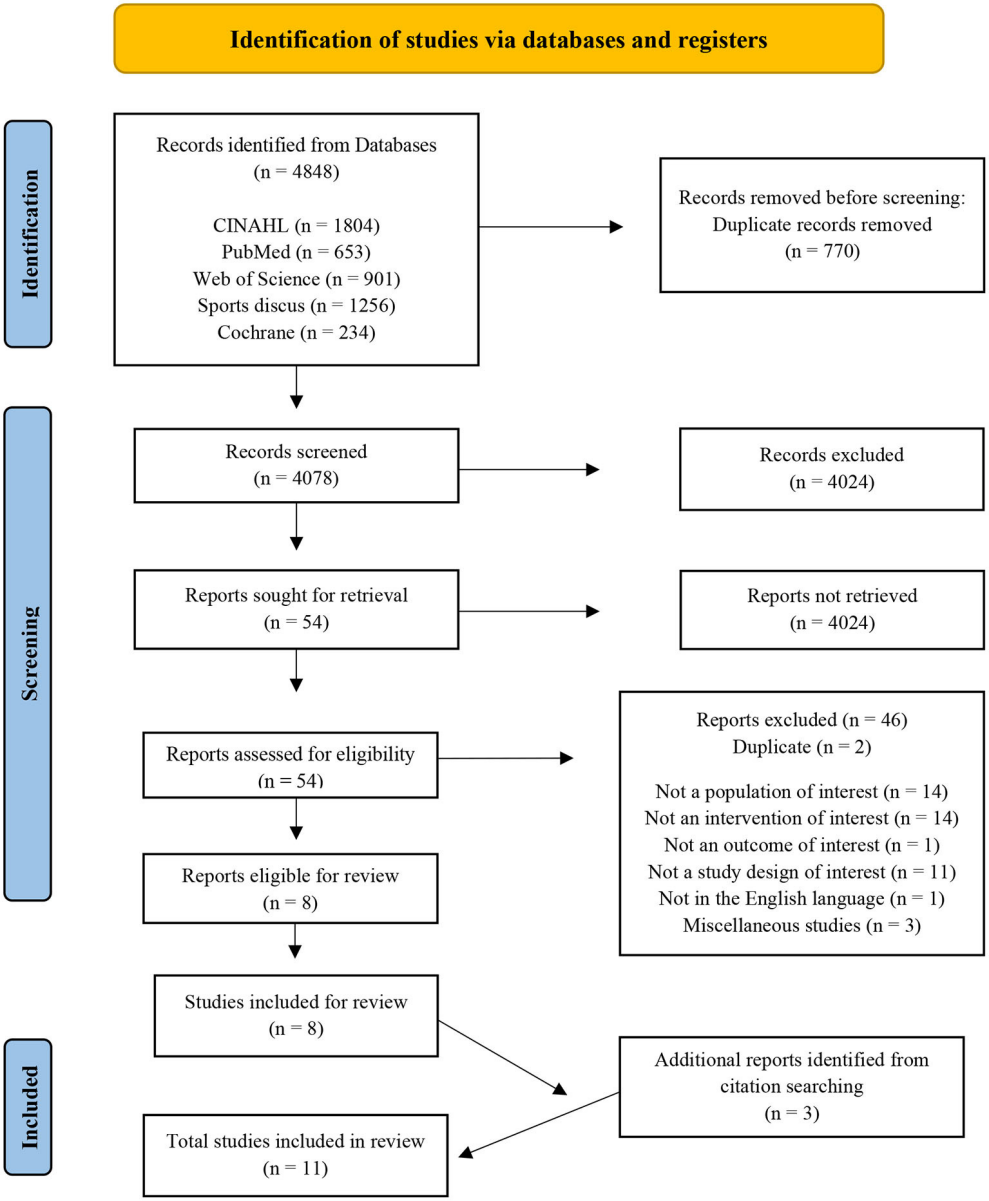
Preferred Reporting Items for Systematic Reviews and Meta-analysis flow diagram of the studies included in the review (48).

The literature search was performed using the electronic databases CINAHL, PubMed, SPORTDiscus, Web of Science, and the Cochrane Library. The search strategy consisted of three phases to create terms following PICOS criteria. The main search concepts were mild Traumatic Brain Injury (mTBI), Nutritional interventions and Recovery outcomes. Phase I, II, and III included database searching, developing and applying key search terms, synonyms, and related terms specific to each concept, which were then refined (Table 1) and employed in the final searches on January 6, 2021, performed by three authors (EF, LR, ED). Citations were exported to an Endnote X9 library, and print screen records were saved for each database (Supplementary material 1). No publication date restrictions were used in the search process. Table 1 presents the final search terms, resulting in a total of 4,848 identified articles of potential interest (Figure 1; PRISMA).

### Eligibility criteria

The eligible articles were required to meet the following inclusion criteria:

(1) Studies involving human(s) (children and adolescents <18 years and adult populations ≥18 years), clinically diagnosed with a concussion/mTBI (with a GCS 13–15), due to a known or reported mechanism.
(2) Study participants received nutrition or a nutritional intervention following injury onset or diagnosis within the acute phase (<14 days) (8, 49).
(3) Published original research [with a study design including retrospective, cross-sectoral, quasi-experimental, parallel, randomized controlled trial (RCT), systematic reviews, observational studies, abstracts (with data), and pilot/feasibility studies].
(4) Measured an outcome of acute recovery [in this review, concussion/mTBI recovery is defined within 10–14 days for adults and 0–28 days for children post-injury onset (28, 50)].
(5) Written in the English language.

### Study screening

The 4,848 identified articles exported to EndNote X9 (Clarivate, USA) were screened (see Figure 1). Duplicate records were identified and removed, screening for study inclusion commenced, based on titles, abstracts, and full papers. During each stage, studies were coded and recoded using a hierarchy of exclusion criteria created by authors per PICOS; (1) duplicate (D), not a (2) population of interest (NP); (3) intervention of interest (NI); (4) outcome of interest (NO); (5) study of interest (NS), (6) English (NE), and (7) miscellaneous (M). Studies that passed the seven criteria were coded INC and included for next stage analysis; those coded “M” also passed for further investigation because the rationale to exclude or include was unclear. The collection, initial screening, and coding according to hierarchy of exclusion was carried out by one researcher (EF) with random checks throughout by two researchers (ED, LR) for consensus on article exclusion and inclusion. The final stage of full paper analyses and coding was conducted by three researchers (EF, LR and ED), ensuring coherence, and minimizing risk of error and bias.

### Data extraction

Data extraction was performed by EF using a modified National Health and Medical Research Council (NHMRC, Australia) template tool for cohort and RCT studies (51). Relevant data were identified as follows and extracted, such as author details, study design, population characteristics, and nutrition/nutritional intervention detail (type, control conditions, timing, sample, dosage and duration), recovery outcome measures employed [return to play (RTP), length of stay (LOS), GCS, symptom resolution, cognitive and balance tests], and noteworthy results from each publication. Study design and nutrition intervention types varied, and data were lacking in three included RCT abstract publications investigating the use of Docosahexaenoic acid (DHA) and/or Eicosapentaenoic acid (EPA), and combined DHA for recovery outcomes post-concussion/mTBI. In some incidences, abstracts did not provide detail on the specific dosing protocols, injury events/mechanisms, sample sizes, sex breakdowns, and adverse outcomes. Therefore, the researcher (EF) supplemented missing abstract data with details from relevant clinical trial registrations (50, 52, 53) where possible. Additionally, the relevant authors were also contacted to request final publication data, given insufficient quantitative data presented in the abstracts. However, publications were not available. Therefore, it was not feasible to perform a meta-analysis with the results due to deficient data and a qualitative analysis was carried out instead.

### Quality assessment and risk of bias

Quality appraisal of all included publications was performed using the Academy of Nutrition and Dietetics Evidence Analysis Library Quality Criteria Checklist (2016) (54). This checklist assessed quality using four relevance and 10 validity type questions. The criteria were independently checked by researchers (EF, LR), and studies were assigned a “yes,” “no” or “NA” response. Each study was required to meet four specific validity questions: use of participant selection, study group comparability, detailed intervention and control conditions, defined outcomes and measures used and any of the other six criteria. Studies were designated either a positive, neutral, or negative rating based on the answers to the 10 questions. Any discrepancy was discussed and resolved by consensus with a third researcher (ED). The quality of each study was considered further during the analysis of results. Quality rating results are presented in Supplementary material 2.

## Results

The PRISMA flow diagram (Figure 1) displays the results of the search. A total of 4,848 citations were identified, screened for eligibility, with 54 studies relevant for full-text reading and evaluation. Eight studies met the full inclusion criteria, containing seven trial interventions and one systematic review, revealing one extra intervention (5). Reference list hand searching provided three additional studies. A total of 11 studies were reviewed, and the final results are presented in Table 2 [Characteristics of studies included (*n* = 11)] and Table 3 (Detail of included studies). Quality assessments resulted in nine “positive (+)” and two “neutral (ø)” studies rated outcomes using the Academy of Nutrition and Dietetics (ADA) checklist (2016).

**Table 2:** Characteristics of the studies included in the review (*p* = 11) including study objective.

**References**	**Setting, country**	**Study type**	**Nutrition intervention**	**Study objective**
**Pediatric populations**				
Miller et al. (55)	Sports medicine at Texas Scottish Rite hospital for children, Texas, United States of America	Randomized double blind, placebo-controlled, feasibility trial	Omega-3, DHA	To determine the feasibility, outcome, and safety of DHA as an early treatment method for sports related concussions in pediatrics
Standiford et al. (56)	Lakelands emergency department, Michigan, United States of America	Randomized cohort trial	Magnesium oxide (PO)	To explore positive potential role for magnesium in improving symptoms following concussion, a type of mild TBI (mTBI)
**Adult populations**				
Abdullah et al. (57)	Surgical hospital ward, Sultanah Nur Zahirah, Kuala, Terengganu, Malaysia	Observational dietary intervention	Calorie and protein intake	To determine baseline calorie and protein intake data, to inform early and appropriate medical nutrition therapy (MNT) planning and administration according to patients' acute and sub-acute requirements post TBI
Bica et al. (58)	East Carolina University, United States of America	Randomized double blind, placebo-controlled trial	Omega-3, DHA	To determine if an early high dose of a Docosahexaenoic acid (DHA) supplement in Division I NCAA American football athletes with diagnosed concussion would decrease their number of days out of competitive sport participation
Bisri et al. (59)	Hasan Sadikin hospital, Bandung, Indonesia	Prospective, randomized single blind, controlled trial	Hyperosmolar sodium lactate (HSL) infusion	To evaluate the effect of exogenous lactate on cognitive function in patients with mTBI
Chen et al. (60)	China's Medical University hospital's neurosurgery/emergency departments, Taiwan, China	Randomized double blind, placebo-controlled, phase II pilot trial	Cerebrolysin nootropic infusion	To investigate how cerebrolysin therapy enhances patients' cognitive recovery post mTBI and determine its efficacy and safety
Falk et al. (61)	Michigan State emergency department, Michigan, United States of America	Randomized double blind, placebo-controlled, clinical pilot trial	Omega-3s, DHA and EPA	To determine if omega-3 fatty acids post mTBI will provide neuroprotection by examining its effect on blood-based biomarkers of inflammation and neurogenesis
Hoffer et al. (5)	United States Active Military Setting, Al Anbar, Iraq	Randomized double blind, placebo-controlled study	*N*-acetyl cysteine (NAC)	To compare the efficacy of NAC vs. placebo on symptoms associated with blast exposure induced mTBI in a combat setting
Lee et al. (62)	Trauma center emergency department, Korea, Asia	Retrospective study (with controlled intervention trial)	Vitamin D	To investigate the effects of long term vitamin D (VD) supplementation on recovery outcomes of patients in the acute phase of a TBI
Zafonte et al. (63)	Level 1 trauma centers, United States of America	Randomized double-blind, placebo-controlled trial	Citicoline nootropic	To update on the current status of the citicoline brain injury treatment
		(design and methods)		(COBRIT) trial, describe the design and rationale post injury (neuroprotective properties)
Zafonte et al. (64)	Level 1 trauma centers, United States of America	Randomized double blind placebo-controlled, phase III trial	Citicoline nootropic	To determine the ability of citicoline to positively affect functional and cognitive status in persons with complicated mild, moderate, and severe TBI

DHA, docosahexaenoic acid; PO, oral administration; TBI, traumatic brain injury; mTBI, mild TBI; MNT, medical nutrition therapy; NCAA, National Collegiate Athletic Association; HSL, hyperosmolar sodium lactate; EPA, eicosapentaenoic acid; NAC, N-acetyl cysteine; VD, vitamin D; COBRIT, citicoline brain injury treatment.

**Table 3:** Detail of included studies (*n* = 11).

**References**	**Study design (type, period), quality rating (+, –, ø)**	**Population (sample size, age, sex, event, injury) GCS score**	**Nutrition intervention (type, dose, duration)**	**Intervention sample sizes**	**Outcome measures (used, timepoints)**	**Key findings**	**Author conclusions, key takeaways**
**Pediatric populations**							
Miller et al. (55)	Randomized double blind, placebo-controlled, feasibility trial of DHA over 12 weeks, Positive (+)	*N* = 40 pediatrics aged 14–18 years, (DHA group x̄ = 16.02 years, placebo group x̄ = 15.92 years), 67.5% (*n* = 27) male, 32.5% (*n* = 13) female Diagnosed with a sports related concussion (SRC), enrolled within 96 h, following sports injury onset, [American Football 42.5% (*n* = 17), Soccer 17.5% (*n* = 7), Volleyball 12.5% (*n* = 5), Wrestling 7.5% (*n* = 3), Basketball 5.0% (*n* = 2), Softball 5.0% (*n* = 2), Lacrosse 5.0% (*n* = 2), Baseball 2.5% (*n* = 1), Karate 2.5% (*n* = 1)]. 70% had a previous history of TBI	Supplement 2,000 mg DHA or 950 mg placebo, Dose *via* 4 × capsules daily, (2 × at breakfast and 2 × at dinner)	DHA: *n* = 20 [70% male (*n* = 14), 30% female (*n* = 6)], placebo: *n* = 20 [65% male (*n* = 13), 35% female (*n* = 7)]	Symptom resolution (SCAT 3, no. days), ImPACT neurocognitive test, RTS progressions, RTS clearance to begin, resolution of balance impairments, using mBESS, compliance, adverse outcomes, and safety of high dose DHA treatment All measured on enrolment, and weeks 1, 2, 4 and 12	Symptom resolution 4 days earlier for DHA, 16.1 days, vs. placebo 20.9 days, (*p =* 0.082) ImPACT neurocgnitive test scores normal for DHA at 12.2 days, vs. 16.8 days for placebo (*p =* 0.382) RTS initiation time sooner for DHA 14.0 (9–39) days, vs. placebo 19.5 (8–66) days Clearance to begin RTP earlier for DHA at 21.4 days, vs. placebo 23.4 days (*p =* 0.115) Rate of follow up and completion was poor in DHA (50%, *n* = 10) vs. placebo (75%, *n* = 15) groups Reasons were not documented but may have been due to symptom resolution; those cleared to RTP before week 4 were told that they could forgo additional visits but attend at week 12 Adverse outcomes ocurred in 10% of DHA group [Rhenyolds (*n* = 1) syndrome and eructation (burping) (*n* = 1)]	2,000 mg of a DHA supplement twice daily for 12 weeks is a feasible and safe early treatment method following SRC in pediatrics. DHA shows that it may allow for faster symptom resolution, and sooner RTP. However, a larger trial with more stringent follow up metrics is needed
						Compliance was similar DHA 61.57% and placebo 66.34% only in those that completed the trial	
Standiford et al. (56)	Two-armed randomized cohort intervention of magnesium oxide (PO) over a 5 day period, Positive (+)	*N* = 17 pediatrics, aged 12–18 years, sexes not told Diagnosed with mTBI, GCS range >13 on arrival to Lakelands emergency department (ED) within 48 h of impact/injury onset	Supplement 400 mg magnesium oxide (PO) and Tylenol 500 mg, treatment, or 500 mg Tylenol only placebo, dose *via* 2 × tablets, twice daily, for 5 days (120 h)	Magnesium 53% (*n* = 9), placebo 47% (*n* = 8)	Return to functional outcomes measured using; PCSS from admin. (0 h) time to 120 h later Compliance and follow up were self-reported or conducted *via* phone calls (cases lost in follow up) Adverse effects of medications and patients' need for further intervention in the ED were also measured	Follow up over 5 day period was low. Half (50%) of those initially enrolled followed up completing the trial [magnesium 53% (*n* = 9), placebo, 47% (*n* = 8)] **↓** follow up due to symptom resolution or significant improvement of symptoms - patients reported - feeling better as a factor for them to ignore treatment plan PCSS from 0 to 48 h for magnesium group improved significantly (*p =* 0.016), vs. placebo group (*p =* 0.08) Magnesium group PCSS had significantly decreased at 48 h vs. placebo group (*p =* 0.016)	800 mg magnesium PO (400 mg, 2 × daily following mTBI onset) over 5 days presented a 50% likelihood of acutely treating concussion (symptomology) in pediatrics Magnesium vs. placebo treatment significantly decreased PCSS at 48 h (*p =* 0.016)
**Adult populations**							
Abdullah et al. (57)	Observational dietary intervention, LOS over 8 days, Positive (+)	*N* = 50 patients aged 20–60 years, 56% male (*n* = 28), 44% female (*n* = 22) 92% diagnosed with mild (*n* = 46), 8% moderate (*n* = 4) TBI, and 90% had a GCS between 14–15 (*n* = 45)	24 h dietary recall combined with self-administered food diary (help memory and counterchecking) recording daily	*N* = 50 patients	Calorie and macronutrient intake specifically protein, during LOS, day 1 to DD (3–8 days)	Calorie and protein intakes during LOS - Day 1; calories were low at 23.1% (~516 kcal) and protein was 14.8% (~18.0 g) On DD (3–8 days) - calorie intake	There was a significant difference in intakes between day 1 and DD for all patients Calorie and protein intakes were below estimated REQ amounts. Median calorie REQ - 2,232 kcal/day (IQR = 1,977–2,403 kcal). Median protein REQ - 121.3 g/day
			intake to capture and analyse patient's food intake – 24 h prior to TBI and from day 1 until day of discharge (DD)			increased to 75.0% (approx. 1,674 kcal) and protein to 61.3% (74.4 g) Average (x̄) intakes during LOS – calories 55.2% and protein 41.3% x̄ macronutrient intake during LOS was - 55.5% CHO/17.3% protein/27.2% fat Calorie and protein intakes, increases were linked with the TBI type, severity (92% mTBI) and DD [Calorie intake - TBI severity, DD (*p =* 0.142), x̄ calorie intake (*p =* 0.017)] [Protein intake -TBI severity, DD (*p =* 0.070), x̄ protein intake (*p =* 0.013)] [Calorie intake for females vs. males was lower ↓, DD (*p =* 0.006)]	(IQR = 108.2–130.0 g). The calorie and protein intakes improved during LOS but were suboptimal and may increase TBI patients' risk to develop malnutrition if no intervention or appropriate MNT is provided during and after hospital stay
Bica et al. (58)	Double blind RCT of high dose omega-3 fish oils to treat SRC, over a 30 day period, Positive (+)	*N* = 55 (89%), eligible NCAA division I student athletes, aged 19.8 ± 1.4 years, 60% male (*n* = 33), 40% female (*n* = 22) Diagnosed with a SRC, enrolled within 24 h, by the East Carolina University sports medicine staff	Supplement 2,200 mg omega-3 DHA (440 mg per capsule), or placebo (corn oil), dose *via* 5 × capsules daily for 30 days	DHA: *n* = 23, 60.9% male (*n* = 14), 39.1% female (*n* = 9), placebo: *n* = 32, 59.4% female (*n* = 13), 40.6% male (*n* = 19)	No. of days within a 30 day timeframe to return to play (RTP), for balance and cognition (using ImPACT and Biodex Biosway) to improve and overall compliance	RTP, was 9.1 ± 2.9 days for DHA and 10.7 ± 4.7 days for placebo Compliance was similar DHA 84.20% (*n* = 19.3), and placebo 83.02% (*n* = 26.5) ImPACT and Biodex Biosway scores were not presented Patients (11%) that did not	Evidence that a 2,200mg omega-3 fish oil supplement dose for 30 days did not reduce RTP time following acute SRC (*p* = 0.397). It is unknown if cognitive and balance outcomes improved
						RTP within 30 days were excluded (DHA, *n* = 6; placebo, *n* = 1) in outcome analysis	
Bisri et al. (59)	Prospective, RCT, during surgery period, Neutral (ø)	*N* = 60 patients aged 18–40 years, 78.3% male (*n* = 47), 21.7% female (*n* = 13) Screened within 24 h of sustaining mTBI; 56.7% GCS of 15 (*n* = 34), and 43.3% GCS of 14 (*n* = 26)	Nutrient infusion of 1.5 ml/Kg BW hyperosmolar sodium lactate (HSL) treatment, or hyperosmolar sodium chloride 3% solution (HSS) control, intravenously 15 min before neurosurgery	HSL: *n* = 30 HSS: *n* = 30	Cognitive function using: Mini Mental State Examination (MMSE) questionnaire (at 24 h, 30 and 90 days) post-surgery GCS assessed and recorded (24 h) post-surgery Adverse outcomes measured throughout intervention Vital signs checked every 5 min during surgery Blood osmolality and sodium levels, evaluated and recorded at baseline and (15, 30 min and 6 h) post-surgery	MMSE scores improved greatly for HSL treatment vs. HSS control group HSL MMSE scores ranged: at baseline 16.00 (13.75–18.00), to 21.00 (18.75–22.00) at 24 h, 25.00 (23.75–26.00) day-30 and 28.00 (27.00–29.00) day 90 post-surgery MMSE scores for HSS control were almost unchanged at 24 h and a little increased 30 and 90 days post-surgery	Findings present that a nutrient infusion of 1.5 ml/Kg BW of HSL during surgery post mTBI improved cognitive function. MMSE scores significantly improved in HSL group than HSS group (*p =* < 0.001)
Chen et al. (60)	Double blind randomized controlled phase II pilot trial, over 12 weeks, Positive (+)	*N* = 32 patients aged 30–75 years, 65.6% male (*n* = 21), 34.4% female (*n* = 11) Screened within 24 h of sustaining mTBI	Nutrient infusion of 30 ml cerebrolysin nootropic treatment or saline control, intravenously for 60 min during a 5 day period	Cerebrolysin: *n* = 17, control: *n* = 15	Cognitive outcomes measured using - Cognitive Abilities Screening Instrument (CASI) and MiniMental Status Examination (MMSE) tools. Patients LOS, RTP, compliance and adverse outcomes All measured at baseline, and weeks 1, 4 and 12	Cerebrolysin treatment provided recovery evidence CASI baseline scores were similar for both groups (*p =* 0.1861) CASI scores improved for cerebrolysin from baseline to week 12 (*p =* 0.0461) CASI scores significantly improved from baseline to week 4 (*p =* 0.005) and 12	Nutrient infusion of a 30 ml cerebrolysin nootropic treatment post mTBI for 5 days improved cognitive function outcomes for patients 3 months post injury and improved their domains of long-term memory and drawing
						Domains: of long-term memory (*p =* 0.0256) at week 4, and drawing at week 4 (*p =* 0.0066), and 12 (*p =* 0.0472) improved for the cerebrolysin group. 100% compliance and no adverse outcomes (*p =* 0.003) for the cerebrolysin group	
Falk et al. (61)	Double blind pilot RCT of omega-3 treatment, over 12 weeks, Positive (+)	*N* = 15 patients aged 18–65 years, of all sexes Admission to the ED within 24 to 120 h post mTBI onset, meeting ACRM definition	Supplement 6,000 mg omega-3 treatment (1,000 mg containing 500 mg DHA and 100 mg EPA) or placebo (olive oil), *via* daily dose *via* 6 × capsules for 4 weeks Followed by 1,200 mg omega-3 or placebo for 8 weeks	Allocation not detailed	Inflammation and neurogenesis measured using Neurofilament Light Chain (NFL) biomarker testing. Adherence and symptom resolution measured using the GOS-E, self-reporting, and a battery of neurocognitive tests	Complete resolution of symptoms reported by 92.8% of patients within 30 days (*n* = 14) Frequent symptoms reported: Headache (100%), Dizziness (85.7% (*n* = 2), did not), Taking longer to think-(85.7% (*n* = 2/15), did not), Nausea (78.6% (*n* = 3/15), did not), Fatigue (78.6%, (*n* = 3/15) did not), Photophobia (50% (*n* = 7/15) did not) Overall symptom resolution took median of 12.5 (IQR 9.0–25.0) days	A high dose of omega-3 (DHA and EPA) for 12 weeks post-TBI a high rate (92.8%) of symptom resolution. However, biomarker outcomes, GOS-E and cognitive test results are needed to confirm findings. Additionally in 2021 it was reported that study ceased in due to poor patient enrolment
Hoffer et al. (5)	Double blind RCT, 7 day period, Positive (+)	*N* = 81 patients aged 18–43 years, 99% male (*n* = 80), 1% female (*n* = 1) Diagnosed with mTBI/concussion, following blast exposure during military	Supplement 500 mg *N*-Acetyl Cysteine (NAC) treatment (tablet); for groups, B + D, or placebo (500 mg tablet); for	NAC groups B + D, *n* = 41, placebo groups A + C, *n* = 40	Symptom resolution (no. days), cognitive (neurophysiological and symptomology) tests (on day 7) and drug compliance (from day 1 to 7) measured	Day 7 symptom resolution was significantly greater for NAC (B + D) than placebo (B + C) groups, [OR = 3.6, (*p =* 0.0062), *R*^2^ = 0.37] Group B, receiving NAC	Day 7 symptom resolution post blast exposure significantly improved for the NAC groups [OR = 3.6, (*p =* 0.006)]. Groups receiving NAC within 24 h had 86% increased chance of symptom resolution, then placebo 42%
		service in Al Anbar, Iraq Two enrolment groups: Early [A (*n* = 31), B (*n* = 29)] within 24 h, and delayed [C (*n* = 9), D (*n* = 12)], within 26–72 h after injury	groups, A + C, receiving daily doses of 4,000 mg for 4 days and 3,000 mg for 3 days			within 24 h of blast had 86% chance of symptom resolution and no reported side effects, vs. early placebo group (A) at 42% Compliance from Day 1 to 7 was 100%, with no adverse effects reported	Indicating that NAC, is a safe pharmaceutical, for patients seen within 72 h of blast exposure event (*N* = 81) by the same clinician investigator at a combat zone NAC also presented to have significantly positive impact on neuropsychological test results, no. of mTBI symptoms experienced, and complete resolution by day 7 Secondary findings, for early treatment, seen within 24 h benefiting neurological measures but not neuropsychological outcomes Early NAC treatment and standard therapy administered by an experienced mTBI care provider resulted in a 87% symptom resolution rate at day 7, compared to 11% for those receiving placebo care from the same provider, beginning therapy 24–72 h after blast exposure
Lee et al. (62)	Retrospective, followed by a controlled 3 month vitamin D intervention, Positive (+)	*N* = 345 patients recruited [80.9% mild moderate TBI (*n* = 279)] *N* = 244 were included to treat TBI with vitamin D (VD) intervention Adults, all ages, 75.8% male (*n* = 185 [53 controls, 132 VD]), 24.2% female [*n* = 59 (11 controls, 48 VD)] TBI Events % (*n* = VD/C):	Retrospective measurement of VD serum levels (25(OH)D^3^) following TBI Supplement prescribed to deficient (<30 ng/ml) patients during acute recovery	VD supplement *N* = 180, control *N* = 64	Performance was measured using the GCS/GOS-E, cognitive function using Mini Mental Status Examinations (MMSE) and Clinical Dementia Rating (CDR), VD serum levels were measured (pre, 1 + 3 months post). Adverse outcomes and morality	On admission 94.8% had deficient VD serum levels, 13.62 ± 9.01 ng/ml (*N* = 327/345) Initial GCS was similar for all groups, and there were no significant correlation between VD serum levels and GOS-E scores (*p =* 0.080), or TBI severities and GOS-E scores [severe (*p =* 0.980),	Findings were not just specific to mTBI, as mild and moderate fell into the same GCS category when segmented. Serum VD levels significantly improved for the VD group from baseline to week 12 (*p =* < 0.0001). Performance (GOS-E) and Cognitive [MMSE (*p =* < 0.042) and CDR (*p* = 0.044)] outcomes for VD significantly improved by week 12 Presenting that VD supplements may improve the performance and cognitive
		45.9% Traffic accident (*n* = 81/31), 13.1% Fall (*n* = 24/8), 31.2% Slip (*n* = 55/21), 8.6% Head collision (*n* = 18/3), 1.2% Assault (*n* = 2/1) Initial GCS measurements were 13.14 ± 2.89 ng/ml VD, and 12.36 ± 3.11 ng/ml control (*p =* 0.070) at arrival to ED	100,000 IU (2,500 μg) cholecalciferol (converted by liver to calcifediol) *via* intramuscular injection, followed by 20 IU alfacalcidol (0.5 mg)/day (form does not require converting by liver) *via* tablet			mild-to-moderate, (*p =* 0.923)] No significant difference between VD serum levels and MMSE (*p =* 0.994), or CDR (*p =* 0.974) scores In 3-month follow up - serum VD levels for 70.7% (*N* = 244/345) were measured VD group had improved - from 14.03 ± 8.68 ng/ml at baseline, to 37.42 ± 12.57 ng/ml at week 12 (*p =* < 0.001) No significant performance improvements in GOS-E scores at 3-months presented in both groups or between TBI severities Cognitive outcomes for MMSE/CDR scores in VD/control groups did not differ at week 1 (*p =* 0.981) MMSE scores were significantly different for VD (24.1 ± 7.3) and control (21.6 ± 8.3) groups at week 12 (*p =* 0.045) MMSE recovery rates were greater in VD (*p =* 0.042) group. CDR, recovery rate were greater in VD (*p =* 0.044) group	outcomes of patients with mild-to-moderate TBI when given during the acute phase post-TBI. In deficient patients, this approach is less feasible during acute recovery post-TBI Therefore, patients with TBI should be treated according to their baseline VD serum levels on a diagnosis
Zafonte et al. (63)	Double blind RCT (design + methods), 90 day period (77% of expected enrolment in 2009), Neutral (ø)	*N* = 800 patients (61.9% of (*n* = 1,292) enrolment goal) Data accounted for 85% patient sample, 85% aged 18–70 years (*n* = 683), 80.5% male (*n* = 551), 19.5% female (*n* = 133) TBI Mechanisms: 47% Motor vehicle (*n* = 322), 6% Struck by vehicle (*n* = 38), 32% Fall (*n* = 216), 10% Assault (*n* = 66), 2% Struck on head by object (*n* = 15), 3% Other (*n* = 22) TBI severity: 29% severe (GCS 3–8), 4% moderate (GCS 9–12) and 67% mild complicated TBI (GCS 13–15)	Supplement 2,000 mg citicoline (500 mg), or placebo dose *via* oral/enteral tablets twice daily over 90 days	Information not present	Functional and cognitive performance outcomes at day 90 using the TBI Clinical Trial Networks Core Battery of: 9 measures [Trails Making test A and B, GOS-E, COWAT, CVLT-II, PSI, Digit Span and Stroop Test (1 and 2)] Secondary tests during day 30, 90, and 180 after trial and survival tests post TBI (included: BSI, self-reported psychological status/wellbeing, DRS, and SWLS) Patient Survival and Safety were measured, and 180 day follow up observed maintained effects of treatment post TBI	Results from July 31st, 2009: 77% of expected patients enrolled (*N* = 800) to end in August 2010. Of all patients screened, 13% were included, 6% were unable to consent, and 6% refused to participate. TBI Severity ranged - 29% severe, 4% moderate and 67% CmTBI with a GCS of 13–15 (*N* = 458/679)	Preliminary data on 2,000 mg citicoline, taken twice daily for 90 days post TBI. 2012 CORBIT full study trial
Zafonte et al. (64)	Double blind RCT, phase III trial, during a 90 day period, Positive (+)	*N* = 1,213 patients (between 2007–2011), aged 18–70 years, 74.5% male (*n* = 903), 25.5% female (*n* = 310) TBI Mechanisms: 49% Motor vehicle (*n* = 594), 5.9% Struck by vehicle (*n* = 72), 31.2% Fall (*n* = 378), 0.6% Sport (*n* = 7), 9.3% Assault (*n* = 113), 4% Other (*n* = 49) TBI Severity: 33.5% severe/moderate (*n* = 406)	Supplement 2,000 mg citicoline (500 mg), or placebo dose *via* oral/enteral tablets twice daily for 90 days	Citicoline *n* = 607, [66.4% CmTBI (*n* = 403)], placebo *n* = 606, [66.6% CmTBI (*n* = 404)]	Functional and cognitive performance outcomes at 90 days using TBI Clinical Trials Networks Core Battery of: 9 measures [Trails Making test A and B, GOS-E, COWAT, CVLT-II, PSI, Digit Span and Stroop Test (1 and 2)] Secondary tests during day 30, 90, and 180 after trial (included: BSI, self-reported psychological	Recovery outcomes at 90 and 180 days post treatment presented no significant differences Day 90 Core Battery test outcomes presented positive GCS-E scores for both groups, 35.4% citicoline (*n* = 262) and 35.6% placebo (*n* = 280) All other Battery tests improvements rates varied: from 37.3% to 86.5% in	Citicoline as a 90 day treatment for patients with C-mTBI or moderate/severe TBI did not result in functional and cognitive improvements This 2012 COBRIT study indicates that citicoline was not superior to placebo as an acute and post-acute therapy for participants with broad range of TBI severities
		and 66.5% C-mTBI (*n* = 807)			status/wellbeing, DRS, and SWLS) Patient Survival and safety were measured. Follow up and maintained effects of treatment observed at day 180 post TBI	citicoline and 42.7% to 84.0% in placebo groups At day 90 groups had no significant outcome differences (*p =* 0.76), OR, 0.98 (95% CI, 0.83–1.15) No significant treatment effect between TBI severity subgroups moderate/severe, (*p =* 0.31), OR, 1.14 (95% CI, 0.88–1.49), and C-mTBI (*p =* 0.12), OR, 0.89 (95% CI, 0.72–1.49) Day 180 group Battery test results did not differ significantly (*p* = 0.13), OR, 0.87 (95% CI, 0.72–1.04). Day 180 results for placebo group presented significantly better then citicoline (*p* = 0.004), OR, 0.72 (95% CI, 0.56–0.91), and treatment X severity interactions in C-mTBI patients *N* = 73 Total Deaths, no significant difference in	
						survival rates (*p =* 0.17). 19% CmTBI patients died (*n* = 14), 42.8% citicoline (*n* = 6) and 57.1% placebo (*n* = 8) groups	

Nutrients/Intervention: DHA, docosahexaenoic acid; EPA, eicosapentaenoic acid; HSL, hyperosmolar sodium lactate; NAC, N-acetyl cysteine; PO, oral administration; C, calorie; CHO, carbohydrate; VD, vitamin D; MNT, medical nutrition therapy.

Population/Setting: SRC, sports related concussion; TBI, traumatic brain injury; mTBI, mild traumatic brain injury; C-mTBI, complicated mild TBI; ED, emergency department; ACRM, American Congress of Rehabilitation Medicine; NCAA, National Collegiate Athletic Association.

Outcome Measures: ImPACT, Immediate Post-Concussion Assessment and Cognitive Testing; mBESS, modified Balance Error Scoring System; RTP, return to play/practice; RTS, return to sport; PCSS, Post-Concussion Symptom Severity scores; IQR, inter quartile range; LFU, low follow up; DD, day of discharge; MMSE, mini mental state examination; CASI, cognitive abilities screening instrument; NFL, neurofilament light chain; OR, odds ratio; GCS, Glasgow Coma Scale; GOS-E, Extended Glasgow Outcome Scale; COWAT, controlled oral word association test; CVLT-II, California verbal learning test II; PSI, Processing Speed Index; BSI, brief symptom inventory; DRS, Disability Rating Scale; SWLS, Satisfaction with Life Scale.

Study Design types: RCT, randomized controlled trial.

Symbols: N, population size; n, sample size/total; x, sample average/mean; p, statistical significance p-value; ↓: decreased.

Unit of measurement: Kg BW, kilogram body weight; REQ, requirement; IU, international units; mg, micrograms; ng, nanograms; mg, microgram; ml, milliliter.

### Characteristics of included studies

Eleven studies investigated nutritional interventions employed post-concussion/mTBI onset during the acute and subacute phases in humans, aiming to improve recovery outcomes (Table 2). Studies were published from Malaysia (57), the United States (55, 56, 58, 61, 63, 64), Indonesia (59), China (60), Iraq (5) and Asia (62), between 2009 and 2020. There were eight publications reporting seven different RCTs (5, 55, 58–61, 64) (full publication); (63) (methods and preliminary results), of which one had a prospective design (59). In addition, there was one randomized cohort trial (56), one retrospective trial with a controlled intervention (62), along with one observational intervention (57). The nutritional interventions studied the supplementation of omega-3 polyunsaturated fatty acids (PUFAs); DHA (55, 58), EPA (61), vitamin D (62), magnesium oxide (56), *N*-acetyl cysteine (NAC) (5) and nootropic citicoline (63, 64), and infusions; hyperosmolar sodium lactate (HSL) (59) and nootropic cerebrolysin (65). The observational study focused on the calorie and protein intakes of patients during their stay in the hospital (57). The studies represent 1,908 patients in total; 1,807 (94.7%) were offered a nutritional intervention, 1,432 were diagnosed with mTBI, and/or had a GCS rating of 13–15. Patients ranged in age from 12 years (56) up to 75 years (60), 73.3% were male (*n* = 1,324), 25% Female (*n* = 451), and 2% (*n* = 32) did not disclose sex breakdown (56, 61). More than half reported that patients sought care from hospital surgical wards, emergency departments and trauma clinics; 79.4% did so within 24 h (59–61, 63, 64), and 43% within 96 h (5, 55, 56). A detailed summary of included studies that implemented nutritional interventions, and outcomes are presented in Table 3.

## Results synthesis - description of nutritional interventions

### Supplementation protocols

#### Omega-3 fatty acids

Three human RCTs investigated omega-3 fatty acids, specifically DHA, to determine its role in acute recovery following a sports related mTBI/concussion in pediatric (55) and adult populations (58, 61) when administered within 24–120 h post-injury onset. Miller et al. (55) trialed a daily dose of 2,000 mg DHA over 12 weeks (*n* = 40); for symptom resolution, sooner commencement, and RTP outcomes following sport-related concussion (SRC). DHA led to positive outcomes for return to sport (RTS) initiation (14 vs. 19.5 days) and RTP initiation (21.4 vs. 23.4 days) for patients compared to placebo. Average compliance was 63.96%, and there was one nontoxic outcome (excessive burping), however follow up was poor in the DHA (50%, *n* = 10) group when compared to the placebo during the 12 weeks. Authors did not specifically identify the reason for poor follow-up on week 12. It could have been because the patients' symptoms resolved early and were cleared for RTP within 4 weeks, resulting in them not following up at week 12. Bica et al. (58) randomly assigned student athletes (*n* = 55) diagnosed with a mTBI; 2,200 mg of DHA [39.1% female (*n* = 9), 60.9% male (*n* = 14)] or a placebo [40.6% female (*n* = 13), 59.4% male (*n* = 19)] treatment for 30 days post-injury. There were seven (55 out of 62 were included) patients excluded from this study as they did not RTP within the anticipated 30 day period. RTP took from 6.2 to 12 days (9.1 ± 2.9 days) for the DHA group and 6 to 15.4 days (10.7 ± 4.7 days) for the placebo group (*p* = 0.397); and the average compliance was 83.61%.

The OPTIMA-TBI pilot intervention by Falk et al. (61) focused on neuroprotective outcomes in patients (*n* = 15) who received an omega-3 combined [1,000 mg (500 mg DHA and 100 mg EPA)] or placebo (olive oil) supplement treatment post mTBI. Patients in the omega-3 treatment group received a tiered dose of 6,000 mg for 4 weeks and 1,200 mg for 8 weeks. Symptom resolution took a median of 12.5 days, and complete resolution occurred within 30 days (92.8%), and no significant side effects were reported. Symptoms reported by patients include headaches, dizziness, difficulty thinking, nausea, fatigue, and phobias. Patients also reported that the number of capsules in each dose was difficult to swallow, creating barriers to their adherence. The OPTIMA-TBI pilot abstract is deficient in group allocation, their sex breakdown, biomarker outcomes, the extended Glasgow outcome scale (GOS-E) and cognitive result details; all of which are needed to evaluate the actual effect of omega-3 fatty acids DHA and EPA in acute mTBI recovery accurately.

#### Vitamin D

Lee et al. (62) performed a retrospective investigation of patients' vitamin D levels in the acute phase post TBI. A total of 94% (*n* = 327) of patients were vitamin D deficient (30 ng/ml), with average serum levels of 13.62 ± 9.01 ng/ml, and 80.87% (*n* = 279) were diagnosed with a mild to moderate TBI. Serum levels were measured 3 months later, in 70.7% (*n* = 244/345) of patients who had received a vitamin D (*n* = 180) or no supplement (*n* = 64) intervention to support TBI recovery. The vitamin D group received 100,000 International units (IU) of cholecalciferol intramuscularly (*via* injection) followed by 20 IU alfacalcidol (*via* tablet) the next day if it was viable. Patients who received vitamin D had significantly improved serum levels from baseline 14.03 ± 8.68 ng/ml to 37.42 ± 12.57 ng/ml at week 12 (*p* = < 0.001). Recovery rates were measured using the GOS-E; in those patients receiving vitamin D, scores had significantly improved (*p* = 0.020) from week 1 (6.75 ± 1.40) to week 12 (7.43 ± 1.09). Patients' cognitive recovery outcomes were measured using the mini-mental state examination (MMSE) and the clinical dementia rating (CDR) tests. Those that received a vitamin D supplement (24.1 ± 7.3) had improved MMSE (*p* = 0.045) scores vs. the control group (21.6 ± 8.3) and presented greater overall rates of recovery over the 12 weeks (*p* = 0.042). CDR scores improved for both groups; patients who received vitamin D progressed from 1.10 ± 1.13 at week 1 to 0.82 ± 1.01 at week 12, and the control group increased from 0.94 ± 1.10 to 0.90 ± 1.23. However, the vitamin D group did present the most effective recovery rate for CDR (*p* = 0.044) scores. Findings were positive, highlighting that vitamin D supplementation following mild to moderate TBI may improve patients' long-term performance and cognitive outcomes.

#### Magnesium oxide

Standiford et al. (56) examined the effects of acute magnesium oxide supplementation post-concussion/mTBI for improving symptoms and resulting in a return to functional baseline time in pediatric adolescents (*n* = 17). Patients were randomly assigned either 400 mg magnesium oxide and Tylenol 500 mg (*n* = 9) or 500 mg Tylenol only as control (*n* = 8), in tablet form twice daily for 120 h/5 days following their mTBI diagnosis. Patients receiving magnesium had greater post-concussive symptom severity (PCSS) score improvements. Patients receiving the magnesium treatment baseline scores went from a mean of 49 [standard deviation (SD) = 23] to 29 (SD = 17) within 1 h (a score reduction of 20 marks); 23 (SD = 18) at 48 h (a further 6 marks); and 7.6 (SD = 9) by the final 120 h (a final 15.4 marks). The placebo treatment baseline scores ranged from; a mean of 48 (SD = 19) to 41 (SD = 26) within 1 h (a score reduction of 7 marks); 28 (SD = 19) at 48 h (a further 13 marks); and 19 (SD = 21) by the final 120 h (a final 9 marks). The most significant symptom score decrease occurred at 48 h for patients in the magnesium (*p* = 0.016) group. Patients follow up was poor; 50% dropped out and disregarded treatment plans due to reported symptom improvements and resolution.

#### N-acetyl cysteine

Hoffer et al. (5) conducted a RCT investigating the potential effects of amino acid derivative *N*-Acetyl Cysteine (NAC) in treating blast induced mTBI outcomes for military patients (*n* = 81). There were two intervention groups: early treatment “B” within 24 h (*n* = 29) and delayed treatment “D” within 72 h (*n* = 12), both receiving a loading 4,000 mg dose of NAC orally within 72 h of the blast exposure, followed by 4,000 mg daily for 4 days. Groups also received 3,000 mg daily for three additional days. The patients in the placebo groups (A and C), were administered 500 mg tablets following the same 7 day protocol as in the NAC groups. The primary outcome was to resolve patients mTBI related symptoms, such as dizziness, hearing loss, headache, memory loss, sleep disturbances and neurocognitive dysfunction by day 7. Compliance was 100% in NAC groups; and 86.2% of the patients who received early treatment had full symptom resolution (*n* = 25), while 13.8% (*n* = 4) had 1–2 symptoms remaining at day 7. Those who received NAC later, 16.7% (*n* = 2) had full symptom resolution, and 83.3% had 1–3 symptoms lingering (*n* = 10) at day 7. Neurophysiological Trail Making Tests A and B (TMTA & B) improved significantly for the NAC (B&D) groups, which was equivalent to age-based norms. The placebo group's scores were poor at day 7 (*p* = 0.05); many patients were symptomatic for a prolonged timeframe, 88.9% of those in group A and 58.1% in group B reported still expiring up to five lingering symptoms (*p* = 0.006). Those receiving late interventions of NAC^+^ (group D, 83.3%) or placebo (group A, 88.9%) also reported expiring up to 3–5 lingering symptoms on day 7. Overall, an early (within 24 h) treatment of NAC (group B) was most effective at reducing “no. of day seven symptoms” compared to placebo (group C, NAC–) or those receiving late (group D, NAC+ or group A, NAC–) intervention within 72 h (*p* = 0.005) following mTBI.

#### Nootropic citicoline

Citicoline, an endogenous nootropic was investigated to provide neuroprotection, facilitate and enhance recovery for those who had sustained a mTBI (63, 64). The two studies included are part of the Citicoline Brain Injury Treatment (COBRIT) multi-center trial, which aims to positively affect functional and cognitive status in patients diagnosed with a Complicated mild (C-mTBI), moderate, or severe TBI using citicoline as a treatment. The 2009 CORBIT publication provides preliminary data on the study rationale, design and methods, and characteristics of the patients enrolled (77%, *n* = 800/1,292) at that timepoint (63). The 2012 publication (64) details phase III of the RCT and includes full patient enrolment and treatment data (*n* = 1,213). Patients were randomized within 24 h, receiving either 2,000 mg of citicoline *via* 500 mg capsules (*n* = 607) or placebo capsules (*n* = 606) twice daily, throughout the 90 day study period. Patients with complicated mTBI (C-mTBI) accounted for 66.6% (*n* = 807) of all cases; of which 66.4% (*n* = 403) received citicoline and 66.6% received the placebo (*n* = 404). Functional status and cognitive performance outcomes were measured using the Clinical Trials Network's Core Battery, which included nine specific tests [TMTA & B, GOS-E, Controlled Oral Word Association Test (COWAT), California Verbal Learning Test II (CVLT-II), Processing Speed Index (PSI) and the Digit Span and Stroop tests (1 and 2)]. These primary and secondary outcome tests were performed on days 30, 90, and 180 following patient's enrolment and randomization. On day 180, additional tests were performed to measure survival rates and treatment results. These tests included the Brief Symptom Inventory (BSI) psychological and wellbeing self-report, the Disability Rating Scale (DRS) and the Satisfaction with Life Scale (SLS). Between days 90 and 180, there were no significant differences in Core Battery test results and recovery outcomes post-treatment for either group, although positive outcomes were measured. GOS-E improved by 35.4% for citicoline (*n* = 180/607) and 35.6% for placebo (*n* = 181/606) group patients with moderate/severe and C-mTBI (90 day follow up). The additional Battery Test scales improved; from 37.3% to 86.5% for citicoline and 42.7% to 84.0% for placebo, presenting no significant differences between groups. Both groups were statistically similar (*p* = 0.76) at the day 90 evaluation [global Odds Ratio (OR), 0.98 (95% CI, 0.83–1.15)]. There was no significant treatment effect for the severity subgroups, specifically C-mTBI [*p* = 0.12; OR, 0.89 (95% CI, 0.72–1.49)]. At day 180 groups Battery Test results did not differ (*p* = 0.13), OR, 0.87 (95% CI, 0.72–1.04). However, *post hoc* day 180 analysis showed that the placebo did significantly better than citicoline (*p* = 0.004), OR, 0.72 (95% CI, 0.56–0.91) when comparing treatment and severity (using GCS) interactions for C-mTBI cases. Serious events were reported (*n* = 316), patients died (*n* = 73), 19% were C-mTBI deaths (*n* = 14) and both groups had similar overall survival rates (*p* = 0.17). The COBRIT study indicates that nootropic citicoline was not a superior acute or post-acute treatment for participants who suffered a broad range of GCS defined TBI severities.

### Nutrient infusions

#### Lactate and cerebrolysin

Two studies investigated the effect of implementing a nutrient infusion within 24 h after a TBI for cognitive recovery. Bisri et al. (59) conducted a prospective RTC using an infusion of hyperosmolar sodium lactate (HSL) in mTBI patients (*n* = 30/60) undergoing emergency neurosurgical procedures. Recovery outcome of cognitive function was of interest following mTBI and was measured using the MMSE questionnaire post-surgery (24 h, day 30 and 90). Groups received a 1.5 ml/kg body weight (BW) dose of HSL 3% or control hyperosmolar sodium chloride 3% solution (HSS) infusion within 15 min of surgery, followed by a maintenance infusion of NaCl 0.9% (dose of 1.5 ml/kg BW/h) during surgery. The HSL group had significantly improved MMSE scores at all measured time points compared to the HSS group (*p* = < 0.001). MMSE scores ranged from 16.00 (13.75–18.00) at baseline, to 21.00 (18.75–22.00) 24 h later, 25.00 (23.75–26.00) on day 30, and 28.00 (27.00–29.00) 90 days post-surgery for the HSL group. In comparison in the HSS control group scores ranged from 16.00 (13.75–18.00) at baseline, to 16.00 (14.00–18.00) 4 h later, 17.50 (15.75–20.00) at day 30 and 18.50 (16.00–21.00) 90 days post-surgery. The HSL infusion increased MMSE scores more rapidly than the HSS (*p* = 0.001), confirming that the 1.5 ml/kg HSL infusion is more effective and improves cognitive function for patients post-mTBI.

Chen et al. (60) conducted a pilot RCT of a noorotrophic cerebrolysin infusion intervention on patients within 24 h after sustaining mTBI (*n* = 32) for 5 days to improve their cognitive function. The intervention group (*n* = 17) received 30 ml of cerebrolysin, and the control group (*n* = 15) received 30 ml of saline both for 60 min daily. Outcomes were measured using the Cognitive Abilities Screening Instrument (CASI) and MMSE at baseline, weeks 1, 4 and 12. There were no significate differences present between CASI (*p* = 0.1861) or MMSE (*p* = 0.1431) initial scores at baseline for both groups. There were significant improvements in CASI scores (*p* = 0.0461) from baseline to four (*p* = 0.005), and 12 (*p* = 0.003) weeks post-mTBI among patients receiving cerebrolysin. In contrast, MMSE scores were not as significant between the groups (*p* = 0.111). CASI outcome mean scores for cerebrolysin were 5.2 (±8.9 SD) at week 1, 13.3 (±14.5 SD) at week 4 and 21.0 (±20.4 SD) at week 12, which was the most significant improvement (*p* = 0.0461). The placebo group mean scores were 1.6 (±6.8 SD) at week 1, 7.9 (±11.6 SD) at week 4 and 7.6 (±12.1 SD) at week 12. CASI domains significantly improved for cerebrolysin, especially from baseline to week 4; (*p* = 0.005) and week 12 (*p* = 0.003). Cognitive domains presented notable improvements for patients in the cerebrolysin group. It was measured that their drawing (*p* = 0.066) during weeks 4 and 12, their long-term memory (*p* = 0.0256), and abstract thinking (*p* = 0.0662) at week 12 (*p* = 0.0472) had all improved.

### Acute caloric and macronutrient intake post mTBI

Abdullah et al. (57) conducted an observational intervention looking at the calorie and macronutrient intakes of patients hospitalized after sustaining a TBI. In order to prevent underfeeding and unsuited dietary management practices, researchers wanted to determine an accurate nutritional baseline requirements to support patients during their length of stay at hospital and recovery post TBI. After sustaining a mild/moderate TBI, the brain enters a hypermetabolic period, energy and nutrient needs heighten and may become compromised. However little known on patients' specific energy and nutritional requirements during this acute period after sustaining an mTBI; therefore, this observational data will help inform early and appropriate Medical Nutrition Therapy (MNT) practices. There were 50 patients enrolled in this intervention; of these, 92% (*n* = 46) had sustained mTBI and, during enrolment, patients performed a 24 h dietary recall and a food intake diary which they continued to keep during their length of stay (LOS) at hospital following injury onset (between 1 to 8 days). Researchers analyzed patients dietary recall data and calculated calorie and protein estimations to that confirmed that medians of 2,232 calories [kcal; Inter Quartile Range (IQR) = 1,977–2,403 kcal] and 121.3 g/day of protein (IQR = 108.2–130.0 g) daily would be adequate for meeting patients' recovery needs. Patients' calorie and macronutrient intake, especially protein, were outcomes of interest on admission and during their LOS (3–8 days). On day 1, patients consumed on average 516 kcal, 23.1% of estimated recommendation (IQR 0.0–53.8) and 18.0 g of protein, 14.8% of estimated recommendation (IQR 0.0–40.6), which was low. By patients' day of hospital discharge day (3–8 days) patients' intakes had increased. Patients reported on average consuming 1,674 kcals, 75.0% of their estimated caloric needs (IQR 64.1–84.5), and 74.4 g of protein, 61.3% of their estimated (IQR 53.6–70.4) requirements. A total of 42% (*n* = 21) of patients were discharged by day 3, while the remaining patients (*n* = 29) were discharged to go home by day 8. Patients' average daily calorie intake during their LOS was 55.2% (IQR 37.2–65.9), their average daily protein intake was 41.3% (IQR 28.2–52.2), and their overall average macronutrient intake was 55.5% carbohydrate, 17.3% protein, and 27.2% fat. These data indicate that the type of TBI a patient sustained, its severity and their day of discharge from hospital were significantly linked to an increased calorie (*p* = 0.017 and protein (*p* = 0.013) intakes. Additionally, calorie intakes differed significantly on day of discharge, for females (*n* = 22) who were consuming fewer calories than males (*p* = 0.006). This observational research indicates that patients' food and nutrient intake during LOS at hospital post mild (92%) and moderate (16%) TBI were suboptimal and, as a result, may increase their risk of malnourishment and poor recovery outcomes, especially if patients do not receive appropriately planned MNT following their TBI injury onset and hospital stay.

## Discussion

This systematic review sought to examine the current evidence on nutrition and nutritional interventions prescribed to humans clinically diagnosed with mTBI during its acute period (<14 days) to facilitate and result in measured outcome(s) of recovery. Included studies reported a diverse range of recovery measures and outcomes for patients during acute recovery periods 0–14 days for adults and 0–28 days for children post mTBI onset (8, 66). Nine out of the 11 included studies reported positive recovery outcomes utilizing nutritional interventions in those that sustained an mTBI (5, 55, 56, 58–62).

Following mTBI, patient needs are unique and therefore they should receive calorie and protein requirements tailored to their injury severity, sex and age to facilitate them in achieving optimal recovery (57). In addition, specific nutritional supplements and infusions administered during the acute period post mTBI onset may have a role in facilitating patients' recovery. In the present review intakes of nutrients and non-nutrients, including omega-3s fatty acids DHA and EPA (55, 58, 61), vitamin D (62), magnesium oxide (56), NAC (5), HSL (59), and nootropic cerebrolysin (60) demonstrated a positive effect on recovery outcomes for patients post mTBI. However, due to the diversity between interventions and the limited number of studies conducted in humans, a meta-analysis was not possible.

Post mTBI, the brain undergoes a sequelae of metabolic events and responds by over consuming energy and nutrients which as a result modifies patients' nutritional demands (18, 19, 21). In addition, during this period patients are often at a greater risk of sub optimal energy and nutrient intakes or may not have the stores to support these modifications further limiting repair and recovery. Consequently, patients' energy and nutrient intake post mTBI, especially in the acute phase, need to be managed to prevent and alleviate deficiency and, as a result, support brain recovery. Abdullah et al. (57) found that patients require tailored nutritional supports optimized to their injury severity [mild (92%) and moderate (16%)], sex and take post-injury limitations into account to facilitate them in meeting calorie and protein requirements post-trauma. In addition to preventing nutrient fluctuations, underfeeding, and unsuitable dietary management, this would facilitate patients' recovery, reducing LOS and malnutrition risk following their hospital discharge post injury (57).

Protein can support in repair, recovery, and metabolic control. Protein is made up of amino acids that play a fundamental role in supporting repair and healing of tissue, to inhibit tissue breakdown and atrophy. In response to mTBI, amino acid uptake accelerates, and so does protein breakdown to increase amino acids if they are not met with adequate nutrition (protein intake). Tissue stores (i.e., skeletal muscle) will be targeted, increasing amino acid availability to meet injury demands resulting in deficiency (65) and tissue atrophy for injured patients. However, an adequate energy and nutrient supply will help prevent this, supporting patients in meeting their acute post injury demands (41), and has been found effective for those who had sustained severe TBI (11, 43, 57). Presently there are no clear recommendations on calorie and protein intakes post mTBI. It is evident that patients post mTBI would benefit from a tailored menu and dietary guidelines to help them eat suitable calorie and nutrient-dense foods during their recovery (57).

Additionally, during this acute period, hormonal processes are disturbed which may impact patients' appetite and digestive function, limiting their ability to intake enough energy and nutrients from calorically and nutrient-dense foods (21). Consequently, the downregulation of digestion, and nutrient absorption in the intestines, may reduce the support available from food and nutrients to meet the brain's repair and recovery needs. In these situations, supplementation may be a helpful option to aid in mTBI recovery. Unlike food, supplements can be implemented quickly in absorbable forms that can work immediately in combination with prescribed rest and RTP protocols and therefore may facilitate patients in meeting their individual needs for optimal recovery outcomes post mTBI. However, it too should be noted that some supplements can contain nutrients in excess doses, which can have a negative effect on metabolism, recovery markers and patients' overall health.

### Acute nutritional interventions to support mTBI recovery

Long chain omega-3 fatty acids DHA and/or EPA, supplemented within 24–120 h post mTBI were shown to facilitate symptom resolution and sooner RTP outcomes in three human trials (55, 58, 61). DHA and EPA are fatty acids obtained through the diet *via* certain fish and algae-based products and supplements (20, 29, 67); and benefit patients in supporting performance, recovery, and reducing illness and injury risks (68, 69). DHA specifically makes up 97% of all fatty acids found in the brain structure, and plays a vital role in brain development, cognitive functioning, and potential brain recovery. Despite this, current dietary intakes are poor amongst populations especially those in Westernized countries and athlete groups, resulting in low DHA levels in the brain, and a greater risk of worsened response and further depletion in the event of mTBI (70). Limited DHA availability will subsequently result in reduced neural protection (increase symptomology, oxidative stress, and inflammation) and cognitive recovery and repair support post mTBI (20, 29, 44, 71).

This review has highlighted the potential of omega-3 fatty acid supplementation to be used following mTBI to positively support acute recovery and prevent against negative outcomes associated with low DHA levels. Symptom resolution post mTBI is a key recovery indicator (8), and was measured specifically in patients who received 2,000 mg of DHA (55) or the combined tiered supplementation protocol of DHA and EPA (61). Symptoms of those that were treated with DHA began to resolve on average 16.1 days post supplementation: 4 days earlier than the placebo group (55). Similarly, patients receiving the combined DHA (500 mg) and EPA (100 mg) supplemental protocol, displayed a greater degree of symptom resolution, within an average of 12.5 days (between 9 to 25 days) after their enrolment, with complete resolution achieved by 92.8% of patients within 30 days (61). DHA supplementation, specifically doses of 2,000 mg (55) and 2,200 mg (58) resulted in a more rapid RTS (average 5.5 days) or RTP within 30 days for patients. Miller et al. (55) presented that the DHA group took an average of 14 days to start RTP, vs. placebo groups taking 19.5 days after enrolment. Bica et al. (58) found that the patients that received 2,200 mg DHA and had RTP within 30 days (*n* = 23/55), their RTP began within 6.2–12 days, and they reported no significantly different outcome improvements within 30 days compared to those that did not return within the 30 day study period. However, this did not represent the true effect that omega-3, DHA protocols had on all patients, as 20% (*n* = 6) were excluded from the results for not recovering or RTP within 30 days (58), and follow up was poor for those that RTP before week 12 (55) due to unknown reasons. In addition, patients' ImPact cognitive function and BioSway balance score were not presented.

In rodent studies, preinjury DHA intake of 2,000 mg (72) and fish oil (DHA and EPA) or enriched feeds (73, 74) resulted in reduced levels of serum neurofilament light (NFL), providing evidence of reduced axonal damage and neuroprotection in animals (rodents) post mTBI (75). Similarly, rodents fed omega-3 enriched diets, or supplements, immediately or 24 h post TBI had positive neurological and cognitive recovery, such as reduced oxidative stress, restored homeostasis, improved blood-brain barrier integrity and plasticity markers outcomes. Neural protective benefits were seen in animals that received both high and low doses, 740 mg/kg/day or 370 mg/day DHA infusion (76), fish oil (DHA and EPA) or an enriched feed of 10 mg/kg or 40 mg/kg (73, 74). However, these findings although promising are difficult to translate into safe human trials with evidence that mirror and confirm therapeutic approaches to facilitate acute mTBI recovery (8). Exact doses would have to be tailored to patients needs identified post injury to meet differing mass, sex, and recovery requirements (21, 72, 77). Omega-3 fatty acids, specifically DHA, could be a candidate for acute recovery in humans following mTBI despite being understudied. At present there is no consensus on omega-3 fatty acid supplementation recommendations, even for athletes playing in high-risk sub concussive sports. To date the only recommendation is from the Academy of Nutrition and Dietetics and it is not too specific to mTBI. It recommends the consumption of 500 mg DHA and EPA daily, *via* eating at least two servings of fish each week (75, 78).

Vitamin D is a fat-soluble vitamin and steroid hormone that plays a lesser-known role in neurodevelopment, neuroprotection, neurofunction and overall brain health (19, 79–81). Therefore, adequate serum vitamin D levels are important especially for those who encounter mTBI. Lee et al. (62) found that 94.8% of mTBI patients (*n* = 327) studied were severely deficient during injury onset, up until 12 weeks after receiving an intramuscular 100,000 IU supplement dose (62, 80). Patients' vitamin D levels had been below 25–30 nmol/L of serum/plasma 25(OH) D^3^ with blood concentrations levels as low as 13.62 ± 9.1 ng/ml (22.82–4.52 ng/ml); which provides evidence that they were deficient (59, 65, 82). Despite this, at the 12 week follow-up the patients who received 100,000 IU of vitamin D had increased serum levels and improvements in GOS-E performance and cognitive MMSE and CDR test scores, exhibiting long-term recovery outcomes. However, this did not occur during the acute recovery (<28 days) period, as many were deficient (94.8%) prior to intervention following mTBI.

Adequate vitamin D levels may have put patients in a more optimal recovery position in the event of mTBI (62), providing neuroprotection and support during the secondary injury and helping mitigate damage; by restoring cellular homeostasis and regulating ionic disturbances, free radical production, inflammation and damage (59, 83). In addition, if vitamin D supplements were administered to those not severely deficient during diagnosis, negative outcomes may have been reduced (59, 83). The deficient patients would be better supported if they received a treatment that replenished their specific vitamin D serum levels in the acute phase to provide neuroprotection and support recovery post mTBI.

Similarly, Sharma et al. (84) found that a 120,000 IU vitamin D dose can reduce inflammatory cytokine production in patients' serum, improve their GCS scores and levels of consciousness post TBI. In addition, evidence also suggests vitamin D may have a synergistic effect with progesterone, providing better recovery (60%) outcomes when taken in combination in humans with severe TBI than the hormone progesterone (45%) consumed as treatment in isolated (85). A combination of vitamin D has been beneficial in rodents for improving cognitive impairments and inflammatory events post TBI (86, 87). Meanwhile a vitamin D deficiency primes patients for poorer recovery post mTBI, leading to unregulated inflammatory and immune responses, reduced neuroprotection (81, 84) and increased risk of cell death (47, 87–89). In addition, deficiency can affect testosterone levels and increase patients post-traumatic stress disorder (PTSD) risk (84), resulting in chronic fatigue outcomes post TBI (80, 90). However, combined vitamin D (40 IU/kg) and progesterone (16 mg/kg) has been found to reduce inflammatory markers (cytokines), decrease brain oedema (by day 3), blood-brain barrier disruption (89) and cell death when tested on TBI models (43, 87). Therefore, the findings of Lee et al. (62) present an evidence-based argument to consider tiered supplementation tailored to patients' baseline vitamin D serum measures as a viable recovery support following mTBI diagnosis.

Magnesium supplementation (400 mg for 5 days) was effective as a post mTBI treatment, resulting in acute symptom improvements, especially 48 h following treatment and injury onset (56). Magnesium is a mineral responsible for cellular and enzymatic function; following mTBI, levels acutely drop in the brain, triggering a cellular crisis. This decline increases patients' risk of prolonged symptomology and poorer recovery outcomes. Therefore, an acute magnesium intake may replenish levels in the brain following mTBI and initiate symptomology improvements for patients, as measured by Standiford et al. (56). Since the patients' magnesium levels were not determined prior to injury or treatment, initial magnesium levels were unknown. Magnesium is primarily stored in body tissue, and for this reason is difficult to quantify from in the blood serum, with as little as 0.3% is found there (91). Regardless, during the cellular crisis (following mTBI), the brain becomes overloaded with Ca^+^ and glutamate, and magnesium levels deplete in the brain tissue, impairing cells' ability to function if levels do not replenish within 24 h (56, 92). This becomes an issue for those with below optimal levels during injury onset as the decline can persist for up to 3 days, and control the extent of metabolic events, oxidative stress, and anti-inflammatory responses post mTBI (92, 93). For this reason, restoring magnesium levels with a dose depending on patients' fluctuating serum levels would support neuroprotection regulating metabolic changes (94). Therefore, a pre-treatment may help reduce initial cellular depletion and prevent poor recovery outcomes (21). Although Standiford et al. (56) had a small sample size (nine intervention groups, total *n* = 17), patients' cognitive function and recovery outcomes improved 48 h after treatment. Therefore, these results suggest magnesium oxide may be a viable treatment option for improving the severity of symptoms and facilitating a quicker recovery and return to function following mTBI.

Additionally, magnesium's neuroprotective role has been consistently demonstrated in animal studies, unlike human interventions, which have been inconsistent due to limited evidence and robust protocols (43, 95). Nevertheless, Li et al. (96) did find that Magnesium Sulfate (MgSO_4_) was effective in improving performance measures (GOS scores) in severe TBI patient studies (96, 97). Dhandapani et al. (97) found MgSO_4_ [intravenous (4,000 mg) and intramuscular (10,000 mg) with continued 5,000 mg intramuscular dose every 4 h over 24 h] helped to improve performance [increased GOS scores (73.3 vs. 40%)], reduced mortality (13.3% vs. 43.3% within 1 month), and reduced brain swelling (29.4 vs. 73.3%) in humans with severe TBI (97).

Hoffer et al. (5) found NAC effective in mitigating acute symptoms experienced by humans' post blast mTBI. Especially for those that had received treatment within 24 h reporting full symptom resolution over seven days (86.2%) compared to placebo or later treatment groups (5). Cognitive function enhanced and significantly improved (TMT, MMSE and CASI test scores), for patients receiving NAC within 24–72 h post mTBI (5). NAC is the *N*-acetyl derivative of L-cysteine, an amino acid; it has a protective prooxidative role as a precursor for the antioxidant glutathione (98). It inhibits reactive oxygen species (ROS) and nitrogen species (RNS) scavenging oxygen-rich free radicals, reducing oxidative damage, cell death, improving blood flow and oxygenation in the brain. Following mTBI intracellular glutathione depletes; NAC replenishes levels which in turn neutralizes ROS, preventing deteriorating outcomes and secondary events (20, 21, 99). A trial on retired professional American football players (100) and animals (101) provides evidence that NAC can improve the brain's functional status; at the tissue and cellular levels, reducing inflammation, oxidative stress and secondary brain injury outcomes (100, 101). However, NAC has a low blood-brain barrier permeability, therefore, this effect during acute mTBI recovery at this present moment is uncertain and is only safely seen to support symptom resolution.

Similar benefits were found in interventions prescribing nutrient infusions resulting in recovery outcomes, restoring patients cognitive function post mTBI. Endogenous or exogenous lactate becomes a primary oxidative substrate used during the energy crisis by injured brain cells. Bisri et al. (59) found HSL (1.5 ml/Kg) effective as an exogenous surgical treatment and during acute mTBI recovery within 24 h, improving patients' cognitive functions (59). Following TBI, the metabolic changes that occur in the brain increase its energy demand and reduce blood flow – creating an anaerobic environment leading to an overproduction of lactate in the cellular crisis. Lactate is an energy waste product, and has emerged as an essential fuel, with a role in brain energy metabolism following TBI onset and subsequent crisis. During the acute period lactate serves as an effective substitute fuel instead of glucose, acting as a neuroprotective fluid decreasing intracranial pressure; and supporting brain energy demands post TBI (35, 102, 103).

In addition, cerebrolysin was found to be effect in facilitating cognitive function recovery in patients when administered intravenously within 24 h post mTBI for 60 min over a 5 day period. Cerebrolysin is a non-nutrient neurotrophic derived from a mixture of pig peptides and amino acids. This treatment significantly improved patients brain functioning (CASI scores) from baseline to weeks 4 and 12 (60). Similarly, Muresanu et al. (104) found that patients' receiving 20–30 ml cerebrolysin intravenously within 48 h presented improved performance scores (GOS) 10 days later (104). However, research has been primarily focused on moderate to severe TBI cases and therefore, doses (ranging from 10 to 50 ml/day), durations (from 10 days to 20 months), and outcome measures [GOS, GOS-E, Modified Rankin Scale (mRS) and electroencephalogram (EEG)] may be too intense to satisfy the needs of patients following mTBI. In addition, the CASI and MMSE tests (60, 105) are more suitable to measure mTBI cognitive function recovery (104, 106, 107). As a result, to prevent future heterogeneity between tests used to measure TBI, Vestor et al. (108) developed an eight-outcome scale measuring; GOS-E, Early Rehabilitation Barthel Index, MMSE, Processing Speed Index, Stroop Color-Word Test, Digit Span, Finger Tapping Test, Color Trails Test, and patients' hospital Anxiety and Depression Scale (108). This approach aimed to provide a more systematic and complete view of patients' outcomes to measure recovery after TBI.

### Limitations

This review was specific to human populations assigned a nutrient or nutritional intervention following their mTBI diagnosis during its acute period (<14 days) to provide support, and result in recovery outcomes. Previous systematic reviews on animal models have reviewed nutritional and dietary interventions and the neurological, cognitive, and molecular outcomes post TBI (8, 40). In this review, the included studies present numerous limitations, specifically study designs, characteristics of participants sustaining mTBI (age groups, sex breakdown, mTBI events, and sporting vs. non-sporting individuals), the timing of interventions, test types used and limited findings and measurement of outcomes in abstract publications. The significant heterogeneity consequently affected the analyses possible and detail on results reported.

Patients in this review were aged between 12 (56) to 75 years (60), 73.3% were male (*n* = 1,324), 25% female (*n* = 451) and 2% (*n* = 32) were unknown as they did not disclose their sex, and had a diagnosis *via* GCS score (between 13 and 15) and/or had a reported mTBI/concussion prior to intervention. Some studies did not give detail on the breakdown of participants by sex (56, 61); however, their clinical trial data did mention that all sexes were included. Males accounted for a high proportion of patients. This bias is common in preclinical trials, for example DHA interventions were performed primarily on male rodents (40, 76). In spite of this, post mTBI females often experience fluctuating outcomes, worsening of symptoms and longer recovery timelines than males due to biological, hormonal, physical and genetic differences (109–111). Females present greater likelihood of comorbid neck injuries in acute concussions due to weaker and physically different neck structures (112). Females' hormones fluctuate cyclically; neuroprotection decreases in the luteal phase (progesterone surge). As a result, it has been found that females' quality of life 1 month post TBI reduces and neurological outcomes worsen (113) than those in the follicular phase, or males – who are not affected by progesterone cycles. Levin et al. (114) found that females aged 35–49 years were more vulnerable to persistent mTBI related cognitive and somatic symptoms 12 months post injury [using the Rivermead post-concussion symptom questionnaire (RPQ)]. These findings highlight the importance of ensuring that mTBI management takes a patient's characteristics, sex, and age into account in addition to their caloric and nutrient requirements.

It is important when comparing study results, to consider the methodological differences present, such as nutrient type and mode variations (supplementation and infusion therapy), dose, length of intervention and outcome measurements throughout populations (mTBI, C-mTBI, non-sporting, sports, military, pediatrics, and adults). There were similarities present in the three included omega-3 fatty acid trials, however data were inadequate and variable. Due to these differences between interventions and the limited number of available human studies (*n* = 11) meeting inclusion criteria, a meta-analysis of results could not be conducted, and publication bias was difficult to account for.

## Future research

Research into more robust nutrient intervention designs similar to those included in this review, using human RCTs, are necessary. These findings suggest that implementing a variety of nutrient therapies, especially omega-3 fatty acids may improve and support patients' acute recovery outcomes following mTBI. This could be achieved through dietary protocols tailored to patient's individual need and, increasing education and awareness on how to eat to support brain health and injury recovery. In addition, resolving nutrient deficiencies in patients, if present, is essential for overall health, especially for those susceptible to TBI (such as players involved in contact sport).

## Conclusion

To date there are no nutritional recommendations for the recovery post mTBI. This systematic literature review has shown the potential for certain nutritional therapies to help with recovery. However, due to the heterogenic nature of the studies and the limited sample sizes found it is not currently possible to make definitive recommendations. Omega-3 fatty acids in particular show great potential. Therefore, future work should seek to implement nutritional interventions with RTP protocols that have higher sample sizes, safe dosages, duration, and use a range of suitable performance and cognitive outcome measures.

## Data availability statement

The datasets presented in this study can be found in online repositories. The names of the repository/repositories and accession number(s) can be found in the article/Supplementary material.

## Author contributions

EF, LR, and ED developed the search strategy, completed the search, data extraction, and analysis. EF prepared the manuscript. LR, ED, and AP reviewed and edited the final draft of the manuscript. All authors contributed to the article and approved the submitted version.

## Conflict of interest

Author AP currently receives partial research salary funding from Sports Health Check charity (Australia). He has previously received partial research funding from the Australian Football League, Impact Technologies Inc. Australia, and Samsung Corporation, and has provided expert testimony to courts on concussion injury. The remaining authors declare that the research was conducted in the absence of any commercial or financial relationships that could be construed as a potential conflict of interest.

## Publisher's note

All claims expressed in this article are solely those of the authors and do not necessarily represent those of their affiliated organizations, or those of the publisher, the editors and the reviewers. Any product that may be evaluated in this article, or claim that may be made by its manufacturer, is not guaranteed or endorsed by the publisher.

## References

[1] World Health Organization. Neurological disorders: Public health challenges. (2006). Available online at: https://apps.who.int/iris/handle/10665/43605 (accessed August 19, 2022).

[2] Cassidy JD Carroll L Peloso PM Borg J von Holst H Holm L . Incidence, risk factors and prevention of mild traumatic brain injury: results of the who collaborating centre task force on mild traumatic brain injury. J Rehabil Med. (2004) 36:28–60. 10.1080/16501960410023732 15083870

[3] Lefevre-Dognin C Cogné M Perdrieau V Granger A Heslot C Azouvi P. Definition and epidemiology of mild traumatic brain injury. Neurochirurgie. (2021) 67:218–21. 10.1016/j.neuchi.2020.02.002 32387427

[4] Lumba-Brown A Yeates KO Sarmiento K Breiding MJ Haegerich TM Gioia GA . Centers for disease control and prevention guideline on the diagnosis and management of mild traumatic brain injury among children. JAMA Pediatr. (2018) 172:e182853. 10.1001/jamapediatrics.2018.2853 30193284 PMC7006878

[5] Hoffer ME Balaban C Slade MD Tsao JW Hoffer B. Amelioration of acute sequelae of blast induced mild traumatic brain injury by *N*-acetyl cysteine: a double-blind, placebo controlled study. PLoS ONE. (2013) 8:e54163. 10.1371/journal.pone.0054163 23372680 PMC3553161

[6] Marchand NE Jensen MK. The role of dietary and lifestyle factors in maintaining cognitive health. Am J Lifestyle Med. (2018) 12:268–85. 10.1177/1559827617701066 32063810 PMC6993093

[7] Patch CS Hill-Yardin EL Lewis M Ryan L Daly E Pearce AJ. The more, the better: high-dose omega-3 fatty acids improve behavioural and molecular outcomes in preclinical models in mild brain injury. Curr Neurol Neurosci Rep. (2021) 21:45. 10.1007/s11910-021-01132-z 34227043

[8] McCrory P Meeuwisse W Dvorak J Aubry M Bailes J Broglio S . Consensus statement on concussion in sport-the 5th international conference on concussion in sport held in Berlin, October 2016. Br J Sports Med. (2017) 51:838–47. 10.1136/bjsports-2017-097699 28446457

[9] Colonetti T Uggioni MLR Ferraz SD Rocha MC Cruz MV Rosa MID . Nutritional interventions in children with brain injuries: a systematic review. Nutrients. (2021) 13:1130. 10.3390/nu13041130 33808118 PMC8066061

[10] O'Donnell K Healy A Burke T Staines A McGettrick G Kwasky A . Traumatic brain injury epidemiology and rehabilitation in Ireland: a protocol paper [preprint, version 1; peer review: 2 approved with reservations]. HRB Open Res. (2021). 10.12688/hrbopenres.13209.1 PMC10579856 37854498

[11] Markovic SJ Fitzgerald M Peiffer JJ Scott BR Rainey-Smith SR Sohrabi HR . The impact of exercise, sleep, and diet on neurocognitive recovery from mild traumatic brain injury in older adults: a narrative review. Ageing Res Rev. (2021) 68:101322. 10.1016/j.arr.2021.101322 33737117

[12] Romeu-Mejia R Giza CC Goldman JT. Concussion pathophysiology and injury biomechanics. Curr Rev Musculoskelet Med. (2019) 12:105–16. 10.1007/s12178-019-09536-8 30820754 PMC6542913

[13] Dewan MC Rattani A Gupta S Baticulon RE Hung Y-C Punchak M . Estimating the global incidence of traumatic brain injury. J Neurosurg. (2019) 130:1080–97. 10.3171/2017.10.JNS17352 29701556

[14] Iverson GL Gardner AJ Terry DP Ponsford JL Sills AK Broshek DK . Predictors of clinical recovery from concussion: a systematic review. Br J Sports Med. (2017) 51:941–8. 10.1136/bjsports-2017-097729 28566342 PMC5466929

[15] Kim S Han SC Gallan AJ Hayes JP. Neurometabolic indicators of mitochondrial dysfunction in repetitive mild traumatic brain injury. Concussion. (2017) 2:CNC48. 10.2217/cnc-2017-0013 30202587 PMC6128012

[16] Verboon LN Patel HC Greenhalgh AD. The immune system's role in the consequences of mild traumatic brain injury (concussion). Front Immunol. (2021) 12:620698. 10.3389/fimmu.2021.620698 33679762 PMC7928307

[17] Shukla D Devi BI. Mild traumatic brain injuries in adults. J Neurosci Rural Pract. (2010) 01:082–8. 10.4103/0976-3147.71723 21808509 PMC3139355

[18] Giza CC Hoa DA. The new neurometabolic cascade of concussion. Neurosurgery. (2014) 75(Suppl 4):S24–33. 10.1227/NEU.0000000000000505 25232881 PMC4479139

[19] Casazza K Swanson E. Nutrition as medicine to improve outcomes in adolescents sustaining a sports-related concussion. Explor Res Hypothesis Med. (2017) 2:1–9. 10.14218/ERHM.2017.00029

[20] Di Pietro V Yakoub KM Caruso G Lazzarino G Signoretti S Barbey AK . Antioxidant therapies in traumatic brain injury. Antioxidants. (2020) 9:260. 10.3390/antiox9030260 32235799 PMC7139349

[21] Walrand S Gaulmin R Aubin R Sapin V Coste A Abbot M. Nutritional factors in sport-related concussion. Neurochirurgie. (2021) 67:255–8. 10.1016/j.neuchi.2021.02.001 33582206

[22] Kim K Priefer R. Evaluation of current post-concussion protocols. Biomed Pharmacother. (2020) 129:110406. 10.1016/j.biopha.2020.110406 32768934

[23] Malik S Alnaji O Malik M Gambale T Rathbone MP. Correlation between mild traumatic brain injury-induced inflammatory cytokines and emotional symptom traits: a systematic review. Brain Sci. (2022) 12:102. 10.3390/brainsci12010102 35053845 PMC8773760

[24] Schimmel SJ Acosta S Lozano D. Neuroinflammation in traumatic brain injury: a chronic response to an acute injury. Brain Circ. (2017) 3:135–42. 10.4103/bc.bc_18_17 30276315 PMC6057689

[25] Shen Q Hiebert JB Hartwell J Thimmesch AR Pierce JD. Systematic review of traumatic brain injury and the impact of antioxidant therapy on clinical outcomes. Worldviews Evid Based Nurs. (2016) 13:380–9. 10.1111/wvn.12167 27243770

[26] Silverberg ND Iaccarino MA Panenka WJ Iverson GL McCulloch KL. Dams-O'Connor K . Management of concussion and mild traumatic brain injury: a synthesis of practice guidelines. Arch Phys Med Rehabil. (2020) 101:382–93. 10.1016/j.apmr.2019.10.179 31654620

[27] Kara S Crosswell H Forch K Cavadino A McGeown J Fulcher M. Less than half of patients recover within 2 weeks of injury after a sports-related mild traumatic brain injury: a 2-year prospective study. Clin J Sport Med. (2020) 30:96–101. 10.1097/JSM.0000000000000811 32132366

[28] Hylin MJ Kerr AL Holden R. Understanding the mechanisms of recovery and/or compensation following injury. Neural Plast. (2017) 2017:1–12. 10.1155/2017/7125057 28512585 PMC5415868

[29] Gupta A Summerville G Senter C. Treatment of acute sports-related concussion. Curr Rev Musculoskelet Med. (2019) 12:117–23. 10.1007/s12178-019-09545-7 30887284 PMC6542872

[30] King JA McCrea MA Nelson LD. Frequency of primary neck pain in mild traumatic brain injury/concussion patients. Arch Phys Med Rehabil. (2020) 101:89–94. 10.1016/j.apmr.2019.08.471 31493383 PMC6930963

[31] King DA Hume PA Hind K Clark TN Hardaker N. The incidence, cost, and burden of concussion in women's rugby league and rugby union: a systematic review and pooled analysis. Sports Med. (2022) 52:1751–64. 10.1007/s40279-022-01645-8 35113388 PMC9325800

[32] Harmon KG Clugston JR Dec K Hainline B Herring S Kane SF . American Medical Society for Sports Medicine position statement on concussion in sport. Br J Sports Med. (2019) 53:213–25. 10.1136/bjsports-2018-100338 30705232

[33] Gutierrez L Folch A Rojas M Cantero JL Atienza M Folch J . Effects of nutrition on cognitive function in adults with or without cognitive impairment: a systematic review of randomized controlled clinical trials. Nutrients. (2021) 13:3728. 10.3390/nu13113728 34835984 PMC8621754

[34] Melzer TM Manosso LM Yau S-Y Gil-Mohapel J Brocardo PS. In pursuit of healthy aging: effects of nutrition on brain function. Int J Mol Sci. (2021) 22:5026. 10.3390/ijms22095026 34068525 PMC8126018

[35] Shaito A Hasan H Habashy KJ Fakih W Abdelhady S Ahmad F . Western diet aggravates neuronal insult in post-traumatic brain injury: proposed pathways for interplay. EBioMedicine. (2020) 57:102829. 10.1016/j.ebiom.2020.102829 32574954 PMC7317220

[36] Spencer SJ Korosi A Layé S Shukitt-Hale B Barrientos RM. Food for thought: how nutrition impacts cognition and emotion. NPJ Sci Food. (2017) 1:7. 10.1038/s41538-017-0008-y 31304249 PMC6550267

[37] Gómez-Pinilla F. Brain foods: the effects of nutrients on brain function. Nat Rev Neurosci. (2008) 9:568–78. 10.1038/nrn2421 18568016 PMC2805706

[38] Sharma B Lawrence DW Hutchison MG. Branched chain amino acids (BCAAs) and traumatic brain injury: a systematic review. J Head Trauma Rehabil. (2018) 33:33–45. 10.1097/HTR.0000000000000280 28060208

[39] Kroshus E Garnett B Hawrilenko M Baugh CM Calzo JP. Concussion under-reporting and pressure from coaches, teammates, fans, and parents. Soc Sci Med. (2015) 134:66–75. 10.1016/j.socscimed.2015.04.011 25917137 PMC4651185

[40] McGeown JP Hume PA Theadom A Quarrie KL Borotkanics R. Nutritional interventions to improve neurophysiological impairments following traumatic brain injury: a systematic review. J Neurosci Res. (2021) 99:573–603. 10.1002/jnr.24746 33107071

[41] Walton SR Malin SK Kranz S Broshek DK Hertel J Resch JE. Whole-body metabolism, carbohydrate utilization, and caloric energy balance after sport concussion: a pilot study. Sports Health. (2020) 12:382–9. 10.1177/1941738120923869 32520660 PMC7787565

[42] Frakes MR. The impact of dietary intake on concussion recovery in division I NCAA athletes. (Electronic Theses Dissertations). 1870 Mississippi, MS: University of Mississippi (2020). Available online at: https://egrove.olemiss.edu/etd/1870 (accessed August 19, 2022).

[43] Lucke-Wold BP Logsdon AF Nguyen L Eltanahay A Turner RC Bonasso P . Supplements, nutrition, and alternative therapies for the treatment of traumatic brain injury. Nutr Neurosci. (2018) 21:79–91. 10.1080/1028415X.2016.1236174 27705610 PMC5491366

[44] Anzalone A Carbuhn A Jones L Gallop A Smith A Johnson P . The omega-3 index in National Collegiate Athletic Association Division I collegiate football athletes. J Athl Train. (2019) 54:7–11. 10.4085/1062-6050-387-18 30645147 PMC6410989

[45] Trojian TH Wang DH Leddy JJ. Nutritional supplements for the treatment and prevention of sports-related concussion-evidence still lacking. Curr Sports Med Rep. (2017) 16:247–55. 10.1249/JSR.0000000000000387 28696987

[46] Ashbaugh A McGrew C. The role of nutritional supplements in sports concussion treatment. Curr Sports Med Rep. (2016) 15:16–9. 10.1249/JSR.0000000000000219 26745164

[47] Lawrence DW Sharma B. A review of the neuroprotective role of vitamin D in traumatic brain injury with implications for supplementation post-concussion. Brain Inj. (2016) 30:960–8. 10.3109/02699052.2016.1147081 27185224

[48] Page MJ McKenzie JE Bossuyt PM Boutron I Hoffmann TC Mulrow CD . The PRISMA 2020 statement: an updated guideline for reporting systematic reviews. BMJ. (2021) 372:n71. 10.1136/bmj.n71 33782057 PMC8005924

[49] Lawrence DW Richards D Comper P Hutchison MG. Earlier time to aerobic exercise is associated with faster recovery following acute sport concussion. PLoS ONE. (2018) 13:e0196062-e. 10.1371/journal.pone.0196062 29668716 PMC5905975

[50] Bica D Armen J. High dose omega-3 fatty acids in the treatment of sport related concussions. Clinicaltrials.gov. National Library of Medicine (US) (2013). Available online at: http://clinicaltrials.gov/show/NCT01814527 NLMidentifierNCT01814527 (accessed August 19, 2022).

[51] NHMRC. A Guide to the Development, Implementation and Evaluation of Clinical Practice Guidelines. National HMR, Council, editor. Canberra, ACT: National Health and Medical Research Council (1999). Available online at: https://www.health.qld.gov.au/__data/assets/pdf_file/0029/143696/nhmrc_clinprgde.pdf (accessed August 19, 2022).

[52] Korley F. OPTIMA-TBI pilot study (OPTIMA). Clinicaltrials.gov. National Library of Medicine (US) (2017). Available online at: https://ClinicalTrials.gov/show/NCT03345550 NLM identifier NCT03345550 (accessed August 19, 2022).

[53] Miller SM. DHA for the treatment of pediatric concussion related to sports injury. Clinicaltrials.gov. National Library of Medicine (US) (2013). Available online at: https://ClinicalTrials.gov/show/NCT01903525 NLM identifier NCT01903525 (accessed August 19, 2022).

[54] Academy Academy of Nutrition and Dietetics Evidence Analysis Manual: Steps in the Academy Evidence Analysis Process. USA (2016). p. 91–7. Available online at: https://www.andeal.org/vault/2440/web/files/2016_April_EA_Manual.pdf (accessed August 19, 2022).

[55] Miller SM Zynda AJ Sabatino MJ Ellis HB Dimeff R. Docosahexaenoic acid (DHA) for the treatment of pediatric sport-related concussion: results of a feasibility trial. Orthop J Sports Med. (2019) 29:165. 10.1177/2325967119S00004

[56] Standiford L O'Daniel M Hysell M Trigger C. A randomized cohort study of the efficacy of PO magnesium in the treatment of acute concussions in adolescents. T Am J Emerg Med. (2020) 44:419–22. 10.1016/j.ajem.2020.05.010 33243533

[57] Abdullah MI Ahmad A Wafa SWWSST Latif AZA Yusoff NAM Jasmiad MK . Determination of calorie and protein intake among acute and sub-acute traumatic brain injury patients. Chin J Traumatol. (2020) 23:290–4. 10.1016/j.cjtee.2020.04.004 32423779 PMC7567897

[58] Bica D Armen J Kulas AS. AMSSM research podium presentations. Cli J Sport Med. (2018) 28:239–48. 10.1097/JSM.0000000000000591

[59] Bisri T UB Fuadi I. Effect of exogenous lactate infusion improved neurocognitive function of patients with mild traumatic brain injury. Asian J Neurosurg. (2016) 11:151–9. 10.4103/1793-5482.145375 27057222 PMC4802937

[60] Chen C-C Wei S-T Tsaia S-C Chen X-X Cho D-Y. Cerebrolysin enhances cognitive recovery of mild traumatic brain injury patients: double-blind, placebo-controlled, randomized study. Br J Neurosurg. (2013) 27:803–7. 10.3109/02688697.2013.793287 23656173

[61] Falk H Korley F. The 3rd Joint Symposium of the International and National Neurotrauma Societies and AANS/CNS Section on Neurotrauma and Critical Care August 11-16, 2018 Toronto, Canada. J Neurotrauma. (2018) 35:A-1–285. 10.1089/neu.2018.29013.abstracts

[62] Lee JM Jeong SW Kim MY Park JB Kim MS. The effect of vitamin D supplementation in patients with acute traumatic brain injury. World Neurosurg. (2019) 126:e1421-e6. 10.1016/j.wneu.2019.02.244 30904798

[63] Zafonte R Friedewald WT Lee SM Levin B Diaz-Arrastia R Ansel B . The citicoline brain injury treatment (COBRIT) trial: design and methods. J Neurotrauma. (2009) 26:2207–16. 10.1089/neu.2009.1015 19803786 PMC2824223

[64] Zafonte RD Bagiella E Ansel BM Novack TA Friedewald WT Hesdorffer DC . Effect of citicoline on functional and cognitive status among patients with traumatic brain injury. JAMA. (2012) 308:1993. 10.1001/jama.2012.13256 23168823

[65] Smith-Ryan AE Hirsch KR Saylor HE Gould LM Blue MNM. Nutritional considerations and strategies to facilitate injury recovery and rehabilitation. J Athl Train. (2020) 55:918–30. 10.4085/1062-6050-550-19 32991705 PMC7534941

[66] Quinn DK Mayer AR Master CL Fann JR. Prolonged postconcussive symptoms. Am J Psychiatry. (2018) 175:103–11. 10.1176/appi.ajp.2017.17020235 29385828 PMC6586466

[67] Singh JE. Dietary sources of omega-3 fatty acids versus omega-3 fatty acid supplementation effects on cognition and inflammation. Curr Nutr Rep. (2020) 9:264–77. 10.1007/s13668-020-00329-x 32621236

[68] Thielecke F Blannin A. Omega-3 fatty acids for sport performance-are they equally beneficial for athletes and amateurs? A narrative review. Nutrients. (2020) 12:3712. 10.3390/nu12123712 33266318 PMC7760705

[69] Tipton KD. Nutritional support for exercise-induced injuries. Sports Med. (2015) 45(Suppl 1):93–104. 10.1007/s40279-015-0398-4 26553492 PMC4672013

[70] Heileson JL Anzalone AJ Carbuhn AF Askow AT Stone JD Turner SM . The effect of omega-3 fatty acids on a biomarker of head trauma in NCAA football athletes: a multi-site, non-randomized study. J Int Soc Sports Nutr. (2021) 18:65. 10.1186/s12970-021-00461-1 34579748 PMC8477477

[71] Barrett EC McBurney MI Ciappio ED. Ω-3 fatty acid supplementation as a potential therapeutic aid for the recovery from mild traumatic brain injury/concussion. Adv Nutr. (2014) 5:268–77. 10.3945/an.113.005280 24829473 PMC4013179

[72] Oliver JM Jones MT Kirk KM Gable DA Repshas JT Johnson TA . Effect of docosahexaenoic acid on a biomarker of head trauma in American football. Med Sci Sports Exerc. (2016) 48:974–82. 10.1249/MSS.0000000000000875 26765633

[73] Bailes JE Mills JD. Docosahexaenoic acid reduces traumatic axonal injury in a rodent head injury model. J Neurotrauma. (2010) 27:1617–24. 10.1089/neu.2009.1239 20597639

[74] Mills JD Hadley K Bailes JE. Dietary supplementation with the omega-3 fatty acid docosahexaenoic acid in traumatic brain injury. Neurosurgery. (2011) 68:474–81. 10.1227/NEU.0b013e3181ff692b 21135750

[75] Armstrong A Anzalone AJ Pethick W Murray H Dahlquist DT Askow AT . An evaluation of omega-3 status and intake in *Canadian elite* rugby 7s players. Nutrients. (2021) 13:3777. 10.3390/nu13113777 34836033 PMC8620970

[76] Zhu W Chi N Zou P Chen H Tang G Zhao W. Effect of docosahexaenoic acid on traumatic brain injury in rats. Exp Ther Med. (2017) 14:4411–6. 10.3892/etm.2017.5054 29075341 PMC5647748

[77] Oliver JM Anzalone AJ Turner SM. Protection before impact: the potential neuroprotective role of nutritional supplementation in sports-related head trauma. Sports Med. (2018) 48:39–52. 10.1007/s40279-017-0847-3 29368186 PMC5790849

[78] Ritz PP Rogers MB Zabinsky JS Hedrick VE Rockwell JA Rimer EG . Dietary and biological assessment of the omega-3 status of collegiate athletes: a cross-sectional analysis. PLoS ONE. (2020) 15:e0228834. 10.1371/journal.pone.0228834 32348305 PMC7190167

[79] Lewis JE Poles J Shaw DP Karhu E Khan SA Lyons AE . The effects of twenty-one nutrients and phytonutrients on cognitive function: a narrative review. J Clin Transl Res. (2021) 7:575–620. 34541370 PMC8445631

[80] Tuovinen S Räikkönen K Holmlund-Suila E Hauta-Alus H Helve O Rosendahl J . Effect of high-dose vs standard-dose vitamin D supplementation on neurodevelopment of healthy term infants. JAMA Network Open. (2021) 4:e2124493. 10.1001/jamanetworkopen.2021.24493 34495336 PMC8427371

[81] Anjum I Jaffery SS Fayyaz M Samoo Z Anjum S. The role of vitamin D in brain health: a mini literature review. Cureus. (2018) 10:e2960. 10.7759/cureus.2960 30214848 PMC6132681

[82] Roth DE Abrams SA Aloia J Bergeron G Bourassa MW Brown KH . Global prevalence and disease burden of vitamin D deficiency: a roadmap for action in low- and middle-income countries. Ann N Y Acad Sci. (2018) 1430:44–79. 10.1111/nyas.13968 30225965 PMC7309365

[83] Prins M Greco T Alexander D Giza CC. The pathophysiology of traumatic brain injury at a glance. Dis Model Mech. (2013) 6:1307–15. 10.1242/dmm.011585 24046353 PMC3820255

[84] Sharma S Kumar A Choudhary A Sharma S Khurana L Sharma N . Neuroprotective role of oral vitamin D supplementation on consciousness and inflammatory biomarkers in determining severity outcome in acute traumatic brain injury patients: a double-blind randomized clinical trial. Clin Drug Investig. (2020) 40:327–34. 32172522 10.1007/s40261-020-00896-5 PMC7224135

[85] Aminmansour B Nikbakht H Ghorbani A Rezvani M Rahmani P Torkashvand M . Comparison of the administration of progesterone versus progesterone and vitamin D in improvement of outcomes in patients with traumatic brain injury: a randomized clinical trial with placebo group. Adv Biomed Res. (2012) 1:58. 10.4103/2277-9175.100176 23326789 PMC3544099

[86] Hua F Reiss JI Tang H Wang J Fowler X Sayeed I . Progesterone and low-dose vitamin D hormone treatment enhances sparing of memory following traumatic brain injury. Horm Behav. (2012) 61:642–51. 10.1016/j.yhbeh.2012.02.017 22570859 PMC3517217

[87] Tang H Hua F Wang J Yousuf S Atif F Sayeed I . Progesterone and vitamin d combination therapy modulates inflammatory response after traumatic brain injury. Brain Inj. (2015) 29:1165–74. 10.3109/02699052.2015.1035330 26083048 PMC4894830

[88] Cekic M Cutler SM Vanlandingham JW Stein DG. Vitamin D deficiency reduces the benefits of progesterone treatment after brain injury in aged rats. Neurobiol Aging. (2011) 32:864–74. 10.1016/j.neurobiolaging.2009.04.017 19482377 PMC3586224

[89] Yang J Wang K Hu T Wang G Wang W Zhang J. Vitamin D3 supplement attenuates blood-brain barrier disruption and cognitive impairments in a rat model of traumatic brain injury. Neuromolecular Med. (2021) 23:491–9. 10.1007/s12017-021-08649-z 33616826

[90] Scrimgeour AG Condlin ML Loban A DeMar JC. Omega-3 fatty acids and vitamin D decrease plasma T-tau, GFAP, and UCH-L1 in experimental traumatic brain injury. Front Nutr. (2021) 8:685220. 10.3389/fnut.2021.685220 34150829 PMC8211733

[91] Razzaque M. Magnesium: are we consuming enough? Nutrients. (2018) 10:1863. 10.3390/nu10121863 30513803 PMC6316205

[92] Sperl A Heller RA Biglari B Haubruck P Seelig J Schomburg L . The role of magnesium in the secondary phase after traumatic spinal cord injury. A prospective clinical observer study. Antioxidants. (2019) 8:509. 10.3390/antiox8110509 31653023 PMC6912766

[93] Cernak I Savic VJ Kotur J Prokic V Veljovic M Grbovic D. Characterization of plasma magnesium concentration and oxidative stress following graded traumatic brain injury in humans. J Neurotrauma. (2000) 17:53–68. 10.1089/neu.2000.17.53 10674758

[94] Arango MF Bainbridge D. Magnesium for acute traumatic brain injury. Cochrane Database Syst Rev. (2008) CD005400. 10.1002/14651858.CD005400.pub3 18843689 PMC13465917

[95] Vonder Haar C Peterson TC Martens KM Hoane MR. Vitamins and nutrients as primary treatments in experimental brain injury: clinical implications for nutraceutical therapies. Brain Res. (2016) 1640(Pt A):114–29. 10.1016/j.brainres.2015.12.030 26723564 PMC4870112

[96] Li W Bai YA Li YJ Liu KG Wang MD Xu GZ . Magnesium sulfate for acute traumatic brain injury *J Craniofac Surg*. (2015) 26:393–8. 10.1097/SCS.0000000000001339 25723660

[97] Dhandapani S Gupta A Vivekanandhan S Sharma B Mahapatra A. Randomized controlled trial of magnesium sulphate in severe closed traumatic brain injury. Indian J Neurotrauma. (2008) 5:27–33. 10.1016/S0973-0508(08)80025-1

[98] Šalamon Š Kramar B Marolt TP Poljšak B Milisav I. Medical and dietary uses of N-acetylcysteine. Antioxidants. (2019) 8:111. 10.3390/antiox8050111 31035402 PMC6562654

[99] Hoffer BJ Pick CG Hoffer ME Becker RE Chiang Y-H Greig NH. Repositioning drugs for traumatic brain injury - N-acetyl cysteine and phenserine. J Biomed Sci. (2017) 24:71. 10.1186/s12929-017-0377-1 28886718 PMC5591517

[100] Bhatti J Nascimento B Akhtar U Rhind SG Tien H Nathens A . Systematic review of human and animal studies examining the efficacy and safety of N-acetylcysteine (NAC) and N-acetylcysteine amide (NACA) in traumatic brain injury: impact on neurofunctional outcome and biomarkers of oxidative stress and inflammation. Front Neurol. (2017) 8:744. 10.3389/fneur.2017.00744 29387038 PMC5776005

[101] Amen DG Wu JC Taylor D Willeumier K. Reversing brain damage in former NFL players: implications for traumatic brain injury and substance abuse rehabilitation. J Psychoactive Drugs. (2011) 43:1–5. 10.1080/02791072.2011.566489 21615001

[102] Arifianto MR. Ma'ruf AZ Ibrahim A Bajamal AH. Role of hypertonic sodium lactate in traumatic brain injury management Asian. J Neurosurg. (2018) 13:971–5. 10.4103/ajns.AJNS_10_17 30459851 PMC6208238

[103] Mason S. A novel, multi-faceted perception of lactate in neurology. Front Neurosci. (2020) 14:460. 10.3389/fnins.2020.00460 32499676 PMC7242720

[104] Muresanu DF Ciurea AV Gorgan RM Gheorghita E Florian SI Stan H . A retrospective, multi-center cohort study evaluating the severity- related effects of cerebrolysin treatment on clinical outcomes in traumatic brain injury. CNS Neurol Disord Drug Targets. (2015) 14:587–99. 10.2174/1871527314666150430162531 25924999

[105] Fiani B Covarrubias C Wong A Doan T Reardon T Nikolaidis D . Cerebrolysin for stroke, neurodegeneration, and traumatic brain injury: Review of the literature and outcomes. Neurol Sci. (2021) 42:1345–53. 10.1007/s10072-021-05089-2 33515100

[106] Álvarez XA Sampedro C Figueroa J Tellado I González A García-Fantini M . Reductions in qEEG slowing over 1 year and after treatment with Cerebrolysin in patients with moderate-severe traumatic brain injury. J Neural Transm. (2008) 115:683–92. 10.1007/s00702-008-0024-9 18273537

[107] Wong GKC Zhu XL Poon WS. Beneficial effect of cerebrolysin on moderate and severe head injury patients: result of a cohort study. Acta Neurochir Suppl. (2005) 95:59–60. 10.1007/3-211-32318-X_13 16463821

[108] Vester JC Buzoianu AD Florian SI Hömberg V Kim S-H Lee TMC . Cerebrolysin after moderate to severe traumatic brain injury: prospective meta-analysis of the CAPTAIN trial series. Neurol Sci. (2021) 42:4531–41. 10.1007/s10072-020-04974-6 33620612

[109] Gupte R Brooks W Vukas R Pierce J Harris J. Sex differences in traumatic brain injury: what we know and what we should know. J Neurotrauma. (2019) 36:3063–91. 10.1089/neu.2018.6171 30794028 PMC6818488

[110] Merritt VC Padgett CR Jak AJ. A systematic review of sex differences in concussion outcome: what do we know? Clin Neuropsychol. (2019) 33:1016–43. 10.1080/13854046.2018.1508616 30618335

[111] Mollayeva T Mollayeva S Pacheco N Colantonio A. Systematic review of sex and gender effects in traumatic brain injury: equity in clinical and functional outcomes. Front Neurol. (2021) 12:678971. 10.3389/fneur.2021.678971 34566834 PMC8461184

[112] Sutton M Chan V Escobar M Mollayeva T Hu Z Colantonio A. Neck injury comorbidity in concussion-related emergency department visits: a population-based study of sex differences across the life span. J Womens Health. (2019) 28:473–82. 10.1089/jwh.2018.7282 30592685 PMC6482894

[113] Wunderle K Hoeger KM Wasserman E Bazarian JJ. Menstrual phase as predictor of outcome after mild traumatic brain injury in women. J Head Trauma Rehabil. (2014) 29:E1–8. 10.1097/HTR.0000000000000006 24220566 PMC5237582

[114] Levin HS Temkin NR Barber J Nelson LD Robertson C Brennan J . association of sex and age with mild traumatic brain injury–related symptoms: a TRACK-TBI study. JAMA Netw Open. (2021) 4:e213046. 10.1001/jamanetworkopen.2021.3046 33822070 PMC8025125

