# Supplementary material for: Nutritional interventions to support acute mTBI recovery
Source: Front Nutr. 2022 Oct 14;9:977728. doi: 10.3389/fnut.2022.977728 (PMC9614271; doi:10.3389/fnut.2022.977728)
Supplement: Supplementary file 1 [file Data_Sheet_1.docx]

**Supplement material 1 –** Full Search Strategy

**PICOS Framework Inclusion and exclusion criteria**

| **Primary review question/aim** | |
| --- | --- |
| In all humans diagnosed with concussion/ mTBI, what nutrition or nutritional interventions have been prescribed during the acute phase (< 14 days) following injury and resulting in recovery outcomes? | |
| **Inclusion criteria** | |
| Population | Humans all ages (children, adolescents <18 years and adult populations >18 years) clinically diagnosed with a concussion/ mTBI (GCS of 13 to 15), due to any known/ reported mechanism. |
| Intervention | Following diagnosis of concussion/ mTBI during the acute phase (< 14 day window), either nutrition/ nutritional intervention are prescribed. For this review the acute phase of injury will be defined as minutes after the event up to and including 7 days post event. All reported concussion/ mTBI mechanisms. |
| Outcomes | Measured concussion/ mTBI recovery < 14 days for adults and < 28 days for children post injury onset. Return to play, return to activity or a clinical diagnosis of recovery. |
| Study design | Published original research, randomised control trial (RTC), systematic reviews. Retrospective data analysis, cross sectional study design, parallel studies, where data meeting the PICO can be extracted. Abstracts (with data) will be included initially. Publications in the English language only. |
| **Exclusion criteria** | |
| Population | Non-human, animals, cells, and models. |
| Intervention | Non nutritional interventions. Preclinical/ animal/ cell interventions. No measured or hazardous protocols in place. |
| Outcomes | No measure of recovery post TBI. |
| Study design | All other study designs. Case reports, editorials, commentary’s, review articles (in the case of systematic reviews if relevant data cannot be extracted or does not meet PICOS), consensus statements, positional statements, and opinion pieces, and non-English publications. |

**Phase I** literature search key terms, synonyms, and related terms.

|  | **Population** | **Intervention** | **Outcomes** |
| --- | --- | --- | --- |
| **Key concept** | **Mild traumatic brain injury** | **Nutritional interventions** | **Recovery outcomes** |
| **Search terms**  Key words/ synonyms/ related terms | **Synonyms**: concussion OR cerebral concussion OR sports related concussion OR SRC OR acute concussion OR postconcussion OR post-concussion OR post-concussion syndrome OR concussion risk OR traumatic brain injury OR mild traumatic brain injury OR tbi OR mtbi OR brain injury OR head injury OR mild traumatic head injury OR mild head injury OR sub concussive injury OR head trauma OR acute tbi OR acute traumatic brain injury OR brain injury OR acquired brain injury OR abi OR head impact OR cortical impact OR neuroimaging biomarkers OR neuropsychological testing OR cognitive function OR sensorimotor deficits OR attentional deficits OR neurobehavioral symptoms OR chronic cognitive impairment OR brain pathology OR cognitive impairment OR neurological mechanisms OR pathophysiological changes OR mild cognitive impairment OR neurobehavioral manifestations OR fluid percussion OR acceleration injury | **Synonyms**: diet OR food OR beverage OR calorie OR macronutrient OR micronutrient OR protein OR carbohydrate OR fat OR supplement OR antioxidant OR vitamin OR mineral OR amino acid OR fatty acid OR glucose OR creatine OR nutri OR nutraceutical OR keto OR omega 3 OR Docosahexaenoic acid OR DHA OR herb | **Synonyms**: recovery OR return to play OR rehabilitation OR restoration OR improvement OR reduced secondary brain damage OR return to play OR return to activity OR rehab OR clinical recovery OR return to sport OR healing |

**Phase II:** Limited search terms - Most relevant key words, preformed on **03.12.2021**

|  | **Population** | **Intervention** | **Outcomes** |
| --- | --- | --- | --- |
| **Key concept** | **Mild traumatic brain injury** | **Nutritional interventions** | **Recovery outcomes** |
| **Search terms**  Limited search terms  Most relevant key words | **Synonyms**:  **AND**  concussion OR mild traumatic brain injury OR mild tbi OR mtbi OR mild brain injury | **Synonyms**:  **AND**  diet OR food OR beverage OR calorie OR macronutrient OR micronutrient OR protein OR carbohydrate OR fat OR supplement OR antioxidant OR vitamin OR mineral OR amino acid OR fatty acid OR glucose OR creatine OR nutri OR nutraceutical OR keto OR omega 3 OR Docosahexaenoic acid OR DHA OR herb | **Synonyms**:  **AND**  recovery OR return to play OR rehabilitation |

**Phase II:** New search terms preformed and tested in chosen databased (PubMed, CINAHL, SPORTDiscus, Web of Science, Science Direct and Cochrane Library) on **03.12.2021**

| **Database** |  |  |
| --- | --- | --- |
| **PubMed** | **Boolean**/**Phrase**: diet OR food OR beverage OR calorie OR macronutrient OR micronutrient OR protein OR carbohydrate OR fat OR supplement OR antioxidant OR vitamin OR mineral OR amino acid OR fatty acid OR glucose OR creatine OR nutri OR nutraceutical OR keto OR omega 3 OR Docosahexaenoic acid OR DHA OR herb AND concussion OR mild traumatic brain injury OR mild tbi OR mtbi OR mild brain injury AND recovery OR return to play OR rehabilitation | Search S1 OR S2  **Hit Total**: 635,  632 (limited to English & Academic journals) |
| **CINAHL** | **Boolean/Phrase:** diet OR food OR beverage OR calorie OR macronutrient OR micronutrient OR protein OR carbohydrate OR fat OR supplement OR antioxidant OR vitamin OR mineral OR amino acid OR fatty acid OR glucose OR creatine OR nutri OR nutraceutical OR keto OR omega 3 OR Docosahexaenoic acid OR DHA OR herb AND concussion OR mild traumatic brain injury OR mild tbi OR mtbi OR mild brain injury AND recovery OR return to play OR rehabilitation | Search S1 OR S2  **Hit Total:** 1,783,  1528(limited English & Academic journals) |
| **SPORTDiscus** | **Boolean/Phrase:** diet OR food OR beverage OR calorie OR macronutrient OR micronutrient OR protein OR carbohydrate OR fat OR supplement OR antioxidant OR vitamin OR mineral OR amino acid OR fatty acid OR glucose OR creatine OR nutri OR nutraceutical OR keto OR omega 3 OR Docosahexaenoic acid OR DHA OR herb AND concussion OR mild traumatic brain injury OR mild tbi OR mtbi OR mild brain injury AND recovery OR return to play OR rehabilitation | Search S1 OR S2  **Hit Total**: 1249,  987 (limited to English & Academic journals) |
| **Web of Science** | **Boolean/Phrase:** diet OR food OR beverage OR calorie OR macronutrient OR micronutrient OR protein OR carbohydrate OR fat OR supplement OR antioxidant OR vitamin OR mineral OR amino acid OR fatty acid OR glucose OR creatine OR nutri OR nutraceutical OR keto OR omega 3 OR Docosahexaenoic acid OR DHA OR herb AND concussion OR mild traumatic brain injury OR mild tbi OR mtbi OR mild brain injury AND recovery OR return to play OR rehabilitation | Search S1 OR S2  **Hit Total**: 891,  851 (limited to English & Academic journals) |
| **Science Direct** | **#1** nutrition OR diet or supplement AND concussion OR mild traumatic brain injury AND Recovery  **#2** nutrition OR diet OR supplement AND concussion OR traumatic brain injury AND recovery OR clinical recovery  **#3** nutrition OR diet OR supplement AND concussion OR traumatic brain injury AND Recovery  **#**4 nutrition OR diet OR supplement AND concussion OR traumatic brain injury AND return to play OR clinical recovery  **#5** nutrition OR diet OR supplement AND concussion OR traumatic brain injury AND return to play OR recovery  **#6** nutrition OR diet OR supplement AND concussion OR mild traumatic brain injury AND return to play OR recovery  **#7** nutrition OR diet OR supplement AND concussion OR mild traumatic brain injury AND return to play OR recovery OR rehabilitation | **Hit Total**: 204  (35 research articles)  **Hit Total**: 230  (38 research articles)  **Hit Total:** 235  (39 research articles)  **Hit Total**: 151  (27 research articles)  **Hit Total**: 153  (27 research articles)  **Hit Total**: 143  (26 research articles)  **Hit Total**: 77  (9 research articles) |
| **Cochrane library Search Strategy** | **Boolean/Phrase:(**concussion OR mild traumatic brain injury OR mild tbi OR mtbi OR mild brain injury) AND (diet OR food OR beverage OR calorie OR macronutrient OR micronutrient OR protein OR carbohydrate OR fat OR supplement OR antioxidant OR vitamin OR mineral OR amino acid OR fatty acid OR glucose OR creatine OR nutri OR nutraceutical OR keto OR omega 3 OR Docosahexaenoic acid OR DHA OR herb) AND (recovery OR return to play OR rehabilitation)"  In Cochrane Reviews, Cochrane Protocols, Trials, Clinical Answers, Editorials, Special Collections (Word variations have been searched) | **S #6**  **Hit Total**: 232 |

**Phase III:** Databases were limited databases search following discussion with supervisor. Science direct **w**ill now not be used to run searches as they are limited to 8x terms(EF, LR & ED made this decision on 03/12/2020).

**Phase IV - Final Search Strategy**

Developed and confirmed by researchers(EF, LR & ED) on the 17.12.2020. )

| **Databases finalised** | **Search Terms** |
| --- | --- |
| *PubMed*  *CINAHL*  *Web of Science*  *SPORTDiscus*  *Cochrane* | concussion OR mild traumatic brain injury OR mild tbi OR mtbi OR mild brain injury |
|  | AND  diet OR food OR beverage OR calorie OR macronutrient OR micronutrient OR protein OR carbohydrate OR fat OR supplement OR antioxidant OR vitamin OR mineral OR amino acid OR fatty acid OR glucose OR creatine OR nutri OR nutraceutical OR keto OR omega 3 OR Docosahexaenoic acid OR DHA OR herb |
|  | AND  recovery OR return to play OR rehabilitation |

A search of finalised databases and keywords was be carried out on the 09.01.2021 and citations exported to Endnote X9 desktop library. A print screen of the search was recorded and saved following each database.

| **Final Database search and record of citations exported** | | | |
| --- | --- | --- | --- |
| **Database** |  | **Search Terms** | **Hits** |
| **PubMed** | #1 | concussion OR mild traumatic brain injury OR mild tbi OR mtbi OR mild brain injury  (6=**1-5) | 22,457 |
|  | #2 | diet OR food OR beverage OR calorie OR macronutrient OR micronutrient OR protein OR carbohydrate OR fat OR supplement OR antioxidant OR vitamin OR mineral OR amino acid OR fatty acid OR glucose OR creatine OR nutri OR nutraceutical OR keto OR omega 3 OR Docosahexaenoic acid OR DHA OR herb  (31=**7-30) | 10,759,668 |
|  | #3 | recovery OR return to play OR rehabilitation  (35=**32-34) | 1,150,293 |
|  | #4 | #1 AND #2 | 3,473 |
|  | #5 | #1 AND #2 AND #3 | 653 |
| **CINAHL** | #1 | concussion OR mild traumatic brain injury OR mild tbi OR mtbi OR mild brain injury (6=**1-5) | 14,431 |
|  | #2 | diet OR food OR beverage OR calorie OR macronutrient OR micronutrient OR protein OR carbohydrate OR fat OR supplement OR antioxidant OR vitamin OR mineral OR amino acid OR fatty acid OR glucose OR creatine OR nutri OR nutraceutical OR keto OR omega 3 OR Docosahexaenoic acid OR DHA OR herb  (31=**7-30) | 1,395,052 |
|  | #3 | recovery OR return to play OR rehabilitation  (35=**32-34) | 561,762 |
|  | #4 | #1 AND #2 | 3,057 |
|  | #5 | #1 AND #2 AND #3 | 1,804 |
| **SPORT Discus** | #1 | concussion OR mild traumatic brain injury OR mild tbi OR mtbi OR mild brain injury  (6=**1-5) | 12,681 |
|  | #2 | diet OR food OR beverage OR calorie OR macronutrient OR micronutrient OR protein OR carbohydrate OR fat OR supplement OR antioxidant OR vitamin OR mineral OR amino acid OR fatty acid OR glucose OR creatine OR nutri OR nutraceutical OR keto OR omega 3 OR Docosahexaenoic acid OR DHA OR herb  (31=**7-30) | 359,886 |
|  | #3 | recovery OR return to play OR rehabilitation  (35=**32-34) | 212,813 |
|  | #4 | #1 AND #2 | 2,207 |
|  | #5 | #1 AND #2 AND #3 | 1,256 |
| **Web of Science** | #1 | concussion OR mild traumatic brain injury OR mild tbi OR mtbi OR mild brain injury  (6=**1-5) | 31,135 |
|  | #2 | diet OR food OR beverage OR calorie OR macronutrient OR micronutrient OR protein OR carbohydrate OR fat OR supplement OR antioxidant OR vitamin OR mineral OR amino acid OR fatty acid OR glucose OR creatine OR nutri OR nutraceutical OR keto OR omega 3 OR Docosahexaenoic acid OR DHA OR herb  (31=**7-30) | 13,805,451 |
|  | #3 | recovery OR return to play OR rehabilitation  (35=**32-34) | 1,370,178 |
|  | #4 | #1 AND #2 | 6,214 |
|  | #5 | #1 AND #2 AND #3 | 901 |
| **Cochrane Library** | #1 | concussion OR mild traumatic brain injury OR mild tbi OR mtbi OR mild brain injury  (6=**1-5) | 2,063 |
|  | #2 | diet OR food OR beverage OR calorie OR macronutrient OR micronutrient OR protein OR carbohydrate OR fat OR supplement OR antioxidant OR vitamin OR mineral OR amino acid OR fatty acid OR glucose OR creatine OR nutri OR nutraceutical OR keto OR omega 3 OR Docosahexaenoic acid OR DHA OR herb  (31=**7-30) | 280,289 |
|  | #3 | recovery OR return to play OR rehabilitation  (35=**32-34) | 1,159,24 |
|  | #4 | #1 AND #2 | 429 |
|  | #5 | #1 AND #2 AND #3 | 171 |
|  | #6 | #6 (concussion OR mild traumatic brain injury OR mild tbi OR mtbi OR mild brain injury) AND (diet OR food OR beverage OR calorie OR macronutrient OR micronutrient OR protein OR carbohydrate OR fat OR supplement OR antioxidant OR vitamin OR mineral OR amino acid OR fatty acid OR glucose OR creatine OR nutri OR nutraceutical OR keto OR omega 3 OR Docosahexaenoic acid OR DHA OR herb) AND (recovery OR return to play OR rehabilitation)  From Cochrane Reviews(160), Cochrane Protocols(20), Trials(53), Special Collections(1) | 234 |

Final count of citations exported to EndnoteX9 for screening stage of the systematic literature review of nutrition or nutritional interventions implemented following concussion/ mild traumatic brain injury(mTBI) in humans during the acute injury phase (< 14 days) of recovery.

| **Database** | **No. Citations Exported** |
| --- | --- |
| **PubMed** | 653 |
| **CINAHL** | 1,804 |
| **SPORTDiscus** | 1,256 |
| **Web of Science** | 901 |
| **Cochrane Library** | 234 |
| Total Exported: | 4848 |

**Phase IV**: Final Search Strategy Developed and confirmed (EF, LR & ED) on the 17.12.2020.

| **Databases finalised** | **Search Terms** |
| --- | --- |
| *PubMed*  *CINAHL*  *Web of Science*  *SPORTDiscus*  *Cochrane* | concussion OR mild traumatic brain injury OR mild tbi OR mtbi OR mild brain injury |
|  | AND  diet OR food OR beverage OR calorie OR macronutrient OR micronutrient OR protein OR carbohydrate OR fat OR supplement OR antioxidant OR vitamin OR mineral OR amino acid OR fatty acid OR glucose OR creatine OR nutri OR nutraceutical OR keto OR omega 3 OR Docosahexaenoic acid OR DHA OR herb |
|  | AND  recovery OR return to play OR rehabilitation |
