# Supplementary material for: Nutritional interventions to support acute mTBI recovery
Source: Front Nutr. 2022 Oct 14;9:977728. doi: 10.3389/fnut.2022.977728 (PMC9614271; doi:10.3389/fnut.2022.977728)
Supplement: Supplementary file 2 [file Data_Sheet_2.docx]

**Supplement material 2:** Quality assessment results based on the American Dietetic Association quality criteria checklist

| **Author, Year** | 1.  Was the research question clearly stated? | | 2.  Was the selection of study subjects/ patients free from bias? | | 3.  Were study groups comparable? | | 4.  Was method of handling withdrawals described? | | 5.  Was blinding used to prevent introduction of bias? | | 6.  Were intervention/therapeutic regimens/ exposure factor/ procedure and any comparison described in detail? Were intervening factors described? | | 7.  Were outcomes clearly defined and the measurements valid and reliable? | | 8.  Was the statistical analysis appropriate for the study design and type of outcome indicators? | | 9.  Were conclusions supported by results with biases and limitations taken into consideration? | | 10.  Is bias due to study’s funding or sponsorship unlikely? | | **Overall appraisal score** | |
| --- | --- | --- | --- | --- | --- | --- | --- | --- | --- | --- | --- | --- | --- | --- | --- | --- | --- | --- | --- | --- | --- | --- |
| Reviewers | R1 | R2 | R1 | R2 | R1 | R2 | R1 | R2 | R1 | R2 | R1 | R2 | R1 | R2 | R1 | R2 | R1 | R2 | R1 | R2 | R1 | R2 |
| Adbullah et al., 2020 | ✔ | ✔ | ✔ | ✔ | ✔ | ✔ | ✔ | ✔ | N/A | N/A | ✔ | ✔ | ✔ | ✔ | ✔ | ✔ | ✔ | ✔ | ✔ | ✔ | + | + |
| Bica et al., 2018 | ✔ | ✔ | ✔ | ✔ | ✔ | ✔ | ✔ | ✔ | ✔ | ✔ | ✔* | ✔* | ✔* | ✔* | ✔* | ✔* | ✖ | ✖ | ✖ | ✖ | **+** | **+** |
| Bisri et al., 2016 | ✔ | ✔ | ✔ | ✔ | 3✔2✖  * | 3✔2✖  * | ✔ | ✔ | 3✔  1✖  * | 3✔  1✖  * | ✔ | ✔ | ✔ | ✔ | ✔* | ✔* | ✔ | ✔ | ✔ | ✔ | **ø** | **ø** |
| Chen et al., 2013 | ✔ | ✔ | ✔ | ✔ | ✔ | ✔ | ✔ | ✔ | ✔* | ✔* | ✔* | ✔* | ✔ | ✔ | ✔ | ✔ | ✔ | ✔ | ✖ | ✖ | **+** | **+** |
| Falk et al., 2018 | ✔ | ✔ | ✔ | ✔ | ✔ | ✔ | ✔* | ✔* | ✔ | ✔ | ✔* | ✔* | ✔* | ✔* | ✔* | ✔* | ✖ | ✖ | ✖ | ✖ | **+** | **+** |
| Hoffer et al., 2013 | ✔ | ✔ | ✔ | ✔ | ✔ | ✔ | ✔ | ✔ | ✔ | ✔ | ✔ | ✔ | ✔* | ✔* | ✔* | ✔* | ✔ | ✔ | ✔* | ✔* | **+** | **+** |
| Lee et al., 2019 | ✔ | ✔ | ✔ | ✔ | ✔ | ✔ | ✔ | ✔ | ✖ | ✖ | ✔* | ✔* | ✔ | ✔ | ✔ | ✔ | ✔ | ✔ | ✔ | ✔ | **+** | **+** |
| Miller et al., 2019 | ✔ | ✔ | ✔ | ✔ | ✔ | ✔ | ✔ | ✔ | ✔ | ✔ | ✔ | ✔ | ✔ | ✔ | ✔ | ✔ | ✖ | ✖ | ✖ | ✖ | **+** | **+** |
| Standiford et al., 2020 | ✔ | ✔ | ✔ | ✔ | ✔* | ✔* | ✔ | ✔ | ✔ | ✔ | ✔* | ✔* | ✔* | ✔* | ✔* | ✔* | ✔ | ✔ | Unclear  ✖ | Unclear  ✖ | **+** | **+** |
| Zafonte et al., 2009 | ✔ | ✔ | ✔ | ✔ | N/A | N/A | N/A | N/A | ✔ | ✔ | N/A | N/A | ✔ | ✔ | ✔ | ✔ | ✔ | ✔ | ✔ | ✔ | **ø** | **ø** |
| Zafonte et al., 2012 | ✔ | ✔ | ✔ | ✔ | ✔ | ✔ | ✔ | ✔ | ✔ | ✔ | ✔ | ✔ | ✔ | ✔ | ✔ | ✔ | ✔ | ✔ | ✔ | ✔ | **+** | **+** |

The American Dietetic Association (ADA) Quality Criteria Checklist for Primary Research, Evidence Analysis Library 2016

Overall appraisal score: If most (six or more) of the answers to the above validity questions are “No,” the report should be designated negative (-). If the answers to validity criteria questions 2, 3, 6, and 7 do not indicate that the study is exceptionally strong, the report should be designated neutral (ø). If most of the answers to the above validity questions are “Yes” (including criteria 2, 3, 6, 7 and at least one additional “Yes”), the report should be designated positive (+).
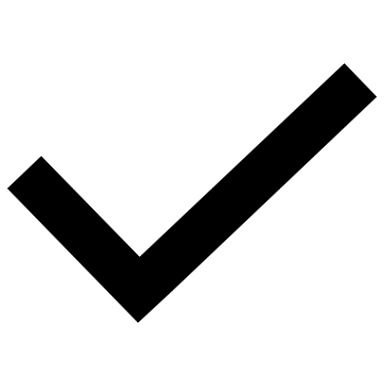
 = yes;
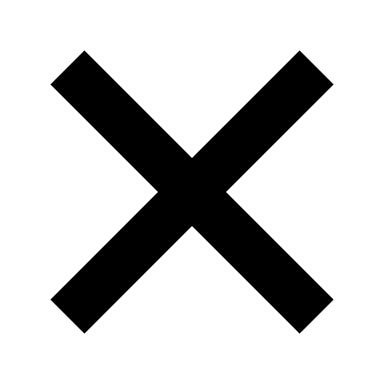
= no.

Those with * include notes below.

| ***Notes on INC studies Quality using ADA** (presneted above with a *) | | |
| --- | --- | --- |
| **Bica et al 2018**   - Q6. Yes (5), No (2) - Q7. Yes (5), No (2) - Q8. Yes (8), No (1) | **Birsi et a 2016**   - Q3. Yes (3), No (2) - Q8. Yes (4), unclear (1) | **Chen et al 2013**   - Q5. Yes (3), No (1)   Q5.2. No   - Q6. Yes (4), No (2) - Q10. unclear - No |
| **Falk et al 2018**   - Q3 Yes (3), unclear (1)   Q3.5 - unclear, as currently in the abstract and clinical pre trial data it is not clear if confounding factors were truly accessed and comparable for 15 subjects.   - Q4.2 - not in abstract/ clinical trial data. - Q6. Yes (4), unclear (2)   Q6.5, 6.6 - unclear in abstract and trial data   - Q. 7.5, 7.6 - unclear in abstract and trial data - Q 8.2 - unclear in abstract and trial data - Q.9 - unclear in abstract and trial data - Q.10 - unclear in abstract and trial data | **Hoffer et al 2013**   - Q3. Yes (4), No (1)   Q3.6. No   - Q6. Yes (5), No (1)   Q6.3. No - exposure, duration could have been > 7 days for a more meaningful effect given recovery may span > 14 days   - Q7 (6, yes; 1 no)   Q7.3. No - short follow up period   - Q8 Yes (5), No (1) - Q9. Yes - Q10. Yes, unclear (1) | **Lee et al 2019**   - Q6. Yes (5), No (1) |
| **Miller et al 2019**   - Q 8.6, 8.7. no – stat analysis in abstract - Q9. No - Q10. No | **Standiford et al 2020**   - Q3. No (1), Yes (3) - Q6. Yes (4), No (1) - Q7. Yes (6), No (1) - researcher feels 5 days may not have been long enough - Q8. Yes (5), No (1) | |
